# Divergence time estimates and the evolution of major lineages in the florideophyte red algae

**DOI:** 10.1038/srep21361

**Published:** 2016-02-19

**Authors:** Eun Chan Yang, Sung Min Boo, Debashish Bhattacharya, Gary W. Saunders, Andrew H. Knoll, Suzanne Fredericq, Louis Graf, Hwan Su Yoon

**Affiliations:** 1 Marine Ecosystem Research Division, Korea Institute of Ocean Science & Technology, Ansan 15627, Korea; 2 Department of Marine Biology, Korea University of Science and Technology, Daejeon 34113, Korea; 3 Department of Biology, Chungnam National University, Daejeon 305-764, Korea; 4 Department of Ecology, Evolution and Natural Resources, Rutgers University, New Brunswick, NJ 08901, USA; 5 Department of Biology, University of New Brunswick, Fredericton, NB E3B 5A3 Canada; 6 Department of Organismic and Evolutionary Biology, Harvard University, Cambridge, MA 02138, USA; 7 Department of Biology, University of Louisiana at Lafayette, Lafayette, LA 70504-3602, USA; 8 Department of Biological Sciences, Sungkyunkwan University, Suwon 16419, Korea

## Abstract

The Florideophyceae is the most abundant and taxonomically diverse class of red algae (Rhodophyta). However, many aspects of the systematics and divergence times of the group remain unresolved. Using a seven-gene concatenated dataset (nuclear EF2, LSU and SSU rRNAs, mitochondrial *cox*1, and plastid *rbc*L, *psa*A and *psb*A genes), we generated a robust phylogeny of red algae to provide an evolutionary timeline for florideophyte diversification. Our relaxed molecular clock analysis suggests that the Florideophyceae diverged approximately 943 (817–1,049) million years ago (Ma). The major divergences in this class involved the emergence of Hildenbrandiophycidae [ca. 781 (681–879) Ma], Nemaliophycidae [ca. 661 (597–736) Ma], Corallinophycidae [ca. 579 (543–617) Ma], and the split of Ahnfeltiophycidae and Rhodymeniophycidae [ca. 508 (442–580) Ma]. Within these clades, extant diversity reflects largely Phanerozoic diversification. Divergences within Florideophyceae were accompanied by evolutionary changes in the carposporophyte stage, leading to a successful strategy for maximizing spore production from each fertilization event. Our research provides robust estimates for the divergence times of major lineages within the Florideophyceae. This timeline was used to interpret the emergence of key morphological innovations that characterize these multicellular red algae.

The Florideophyceae is the most taxon-rich red algal class, comprising 95% (6,752) of currently described species of Rhodophyta^1^ and possibly containing many more cryptic taxa^2^. Florideophytes are relatively well known to the public because they are common along many shorelines and provide economically important cell wall-derived compounds such as agar and carrageenan. The Florideophyceae is equally well known to biologists, in part because of its characteristic (mostly): i) triphasic life cycle consisting of a carposporophyte, gametophyte and tetrasporophyte, ii) the presence of pit-plugs between adjacent cells, and iii) postfertilization cell-cell fusion mechanisms^3^.

Carposporophytes may produce thousands of diploid carpospores from a single syngamy taking advantage of cell-to-cell fusions that occur between the fertilized carpogonium and auxiliary cells. Each carpospore develops into a tetrasporophyte that produces four haploid tetraspores per diploid cell through meiotic division. These additional carposporophyte and tetrasporophyte phases are thought to be evolutionary innovations that explain the success of the red algae. There are, however, a few exceptions to a triphasic life cycle including the ‘asexual tetrasporophytes’ of the Hildenbrandiales, the derived diphasic life cycle in Palmariales, and abbreviated life cycles (i.e., lacking a free-living tetrasporophyte) in some Acrochaetiales^4^, Nemaliales, and Gigartinales^5^.

Pit connections linking neighboring cells are one of the diagnostic features characterizing red algal orders. Diverse combinations of pit connection compartments (i.e., plug core with different number of cap layers and membranes) with molecular data have been used to define the ordinal boundaries of the Florideophyceae. In addition, comparison of the developmental pathway of reproductive structures in male and female gametophytes and tetrasporophytes have also been used to study the diversification of red algal genera and families. Recent phylogenetic studies based on molecular data have resulted in a revised classification system that recognizes 29 orders in five subclasses: Ahnfeltiophycidae, Corallinophycidae, Hildenbrandiophycidae, Nemaliophycidae, and Rhodymeniophycidae^1^,^6^,^7^,^8^. Although previous molecular studies have produced robust phylogenies for relationships among some Florideophyceae^6^, deep relationships among and within other subclasses remain poorly resolved^9^, and the evolutionary timeline for florideophyte divergence has rarely been studied^8^,^10^.

Two important sets of Proterozoic fossils constrain the early evolutionary history of the red algae. The first is commonly considered to be the oldest known taxonomically resolved eukaryotic fossil, the ca. 1,250–1,100 million years old (Ma) *Bangiomorpha pubescens* from the Hunting Formation, Arctic Canada^11^ (see review of *Bangiomorpha pubescens* age constraints^12^). The second consists of anatomically preserved florideophyte fossils from the ca. 580 (635–551) Ma, Doushantuo Formation, southern China, that exhibit growth forms and features closely resembling reproductive structures of modern corallines^13^,^14^,^15^. Based on Doushantuo fossils^13^, the split between the Bangiophyceae and Florideophyceae must have occurred during the Neoproterozoic Era, or earlier. Using these two fossils as calibration points in a multigene phylogenetic analysis, Yoon *et al.*^10^ suggested that the first red alga originated approximately 1,500 Ma, and the Florideophyceae evolved approximately 800 Ma. In another study considering red algal fossil data, Saunders and Hommersand^6^ summarized available data that were consistent with the major lineages of florideophytes diverging before 600–550 Ma, at the end of the Proterozoic Eon. Both studies, however, relied on a limited sampling of florideophyte taxa, and divergence times remain uncertain for the major lineages within the class. Aguirre *et al.*^16^ studied coralline red algal phylogeny and divergence times based on fossil records; well-preserved coralline skeletons in Mesozoic and Cenozoic sedimentary rocks include species placed within the Sporolithaceae (136–130 Ma), Hapalidiaceae (115–112 Ma), and Lithophylloideae (65.5–61.7 Ma), providing additional calibration points for molecular clock analysis^16^,^17^.

Estimating divergence times using molecular data and fossil constraints can considerably advance the evolutionary study of florideophytes and algae in general^10^. To estimate divergence times associated with the Florideophyceae and its constituent subclasses, we performed phylogenetic and molecular clock analyses of combined data (nucleotide sequence of rRNA + amino acid sequence of coding DNA sequence, CDS) from three nuclear (EF2, LSU, and SSU), one mitochondrial (*cox*1), and three plastid (*psa*A, *psb*A, and *rbc*L) genes from 27 florideophycean orders (missing only 2 orders: Entwisleiales and Pihiellales). We generated 180 new sequences consisting primarily of three plastid genes and subsequently compiled a dataset with previously published sequences (mainly EF2, LSU, SSU, and *cox*1) from GenBank.

In order to estimate the divergence time of the Florideophyceae from other red algae, we used seven constraints (Fig. 1, see the methods for details): three red algal fossil dates, (a) 1,222–1,174 Ma for a stem taxon; i.e. the filamentous and spore-bearing red alga *Bangiomorpha*^11^,^12^, (b) 633–551 Ma for Doushantuo fossil-coralline^13^,^14^,^15^ algae, and (c) Cenozoic corallines^16^,^17^ (c1–c2). Four published divergence dates for land plants^18^ were also used as constraints, including (d) 471–480 Ma for the divergence between liverworts (*Marchantia*) and vascular plants, (e) 410–422 Ma for the divergence time between ferns (*Psilotum*) and seed plants, (f) 313–351 Ma for the divergence time between gymnosperms (*Pinus*) and angiosperms, and (g) 138–162 Ma for the monocot-eudicot split (*Zea* and *Arabidopsis*). Divergence times were estimated using Bayesian relaxed-clock methods^19^,^20^. The results are discussed in light of key morphological transitions, such as the origin of the triphasic life cycle and of a diversity of fertilization and diploidization modes in the Florideophyceae.

## Results and Discussion

### Phylogeny of the Florideophyceae

**Figure 1:**
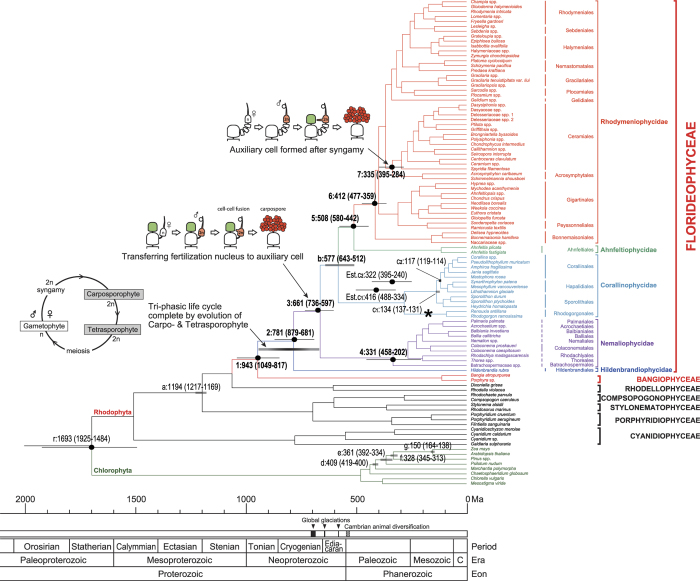
Divergence time and evolution of the Florideophyceae. Estimated times of major divergences based on multigene relaxed clock analysis using the best RAxML tree. Branch lengths are proportional to divergence times (i.e., millions of years ago, Ma). Labels on the node refer to the same splits shown in Supplementary Fig. S1. Seven major divergence times (nodes ‘1–7’) and seven constraints (nodes ‘a–g’) are indicated by mean and 95% HPD (horizontal bar) in parenthesis. The node ‘r’ refers to the root time of the Plantae (i.e., red and green algae). The ‘Est.c1’ and ‘Est.c2’ are estimated times of nodes ‘c1 and c2’ without constraint age, respectively. The three key evolutionary events are indicated with diagrams at the best estimate time frame (arrows). Complete triphasic life cycle of red algae accomplished by the evolution of carposporophyte and tetrasporophyte between time frame of nodes ‘1’ and ‘3’. On the female gametophyte, the fertilization nucleus in the carpogonium (fertilized egg) moved to an auxiliary cell by various so-called ‘cell-to-cell fusion’ mechanisms followed by carposporophyte development and sporic meiosis. This feature is commonly found in most of the florideophycean red algae (except the Hidenbrandiophycidae and Corallinophycidae) and may have evolved at the time of node ‘3’. Asterisk (*) indicates loss of cell-to-cell fusion in the Corallinophycidae. Formation of an auxiliary cell after fertilization (syngamy) is a unique feature of the Ceramiales shown as node ‘7’. The geologic timeline is given under the chronological timeline in a million year scale. Three global glaciations were hypothesized to have occurred 716–670, 645–635, and 581–579 Ma (three arrowheads in the Neoproterozoic Era). The Cambrian animal diversification occurred approximately 520–543 Ma, at the beginning of the Paleozoic Era.

The maximum likelihood (ML) phylogeny inferred from the seven-gene concatenated dataset is shown in Fig. 1 (see also Supplementary Fig. S1). The ML topology was congruent with the Bayesian tree. The phylogeny resolved a monophyletic lineage including the Florideophyceae and Bangiophyceae (node ‘1’ in Fig. 1 and Supplementary Fig. S1) with 60% ML bootstrap (MLB) and 1.0 Bayesian posterior probability (BPP) supports. Within the Florideophyceae, five strongly supported (100% MLB and 1.0 BPP in Supplementary Fig. S1) groups were recovered, equivalent to the five subclasses of Florideophyceae: Ahnfeltiophycidae, Corallinophycidae, Hildenbrandiophycidae, Nemaliophycidae, and Rhodymeniophycidae. These five lineages were recognized in previous studies on the basis of ultrastructural attributes and multigene phylogenetic analyses^7^,^9^,^21^. Therefore, it is highly likely that they reflect accurately the subgroups of Florideophyceae.

The subclass Hildenbrandiophycidae diverged first within the Florideophyceae (node ‘2’ in Fig. 1; 100% MLB and 1.0 BPP in Supplementary Fig. S1) with its deep position consistent with previous studies based on ultrastructural and molecular data^7^,^9^,^22^,^23^,^24^. The sole order in this subclass is the Hildenbrandiales, characterized by pit plugs with a single cap layer covered by a membrane^25^. In contrast, the earlier diverging Bangiales (Bangiophyceae) have pit plugs with a cap but no membrane, and the pit plugs of Compsopogonophyceae represent the ancestral type consisting simply of a plug core, but lacking both a cap and membrane^26^. The Hildenbrandiales includes two genera, *Hildenbrandia* and *Apophlaea,* which form crustose thalli or extensive crusts with upright portions. This order is characterized by the vegetative phase having abundant secondary pit connections that link neighbouring cells. This is atypical in being established without conjunctor cell formation^27^. Zonately and irregularly dividing tetrasporangia have been reported^24^, but it has not been established whether they form meiotically or mitotically^27^. There are no reports of recognizable gametophytic reproductive structures (e.g., eggs, referred to as carpogonia, or spermatangia) or a sexual life history for hildenbrandialean species. Based on the current phylogenetic reconstructions (Supplementary Fig. S1) it is equally likely that the ancestor of Hildenbrandiophycidae was either of the biphasic type that characterizes other earlier diverging clades of red algae (e.g., Bangiophyceae and Rhodellophyceae) or the triphasic type characteristic of the remaining florideophyte subclasses. The only certainty is that the triphasic pattern evolved somewhere between the divergence of the Bangiophyceae and Florideophyceae (node ‘1’ in Fig. 1), and the divergence of the Nemaliophycidae and Ahnfeltiophycidae-Corallinophycidae-Rhodymeniophycidae clade (node ‘3’ in Fig. 1).

The monophyly of the Nemaliophycidae is strongly supported (node ‘4’ in Fig. S1; 100% MLB and 1.0 BPP in Supplementary Fig. S1), consistent with previous studies^7^,^9^,^21^,^28^,^29^. The Nemaliophycidae is characterized by the presence of pit plugs with two cap layers^25^, which likely evolved from pit plugs with a single cap layer (e.g., Bangiales and Hildenbrandiales)^25^,^26^,^29^. Interordinal relationships within the Nemaliophycidae, however, were not resolved. The Batrachospermales and Thoreales were positioned deep in the lineage with moderate support (68% MLB and 1.0 BPP in Supplementary Fig. S1); in contrast, phylogenetic relationships among the Acrochaetiales, Balbianiales, Balliales, Colaconematales, Nemaliales, Palmariales, and Rhodachlyales were weakly supported. We were unable to add representatives of the Entwisleiales to this study, therefore broader taxon sampling with additional sequence data may resolve these relationships.

The Corallinophycidae was positioned between the Nemaliophycidae and Ahnfeltiophycidae-Rhodymeniophycidae clade. This subclass is characterized by pit plugs with a domed outer cap layer and thalli that are mineralized due to calcite precipitation^6^,^21^,^30^. The distinctiveness of the Corallinophycidae is consistent with previous molecular and morphological studies^7^,^9^,^29^,^31^,^32^. This group includes four orders: the Corallinales, Hapalidiales, Rhodogorgonales, and Sporolithales. The Rhodogorgonales is positioned deepest in this lineage (100% MLB and 1.0 BPP in Supplementary Fig. S1) followed by the divergence among the distinctive Sporolithales, Corallinales and Hapalidiales (node ‘C1, and C2, respectively’ in Supplementary Fig. S1; 100% MLB and 1.0 BPP). Relationships among orders and families within the subclass were congruent with previous studies^16^,^17^,^31^,^32^.

The Ahnfeltiophycidae is sister to the Rhodymeniophycidae at node 5 (Fig. 1; 100% MLB and 1.0 BPP in Supplementary Fig. S1). The sister relationship of these two subclasses was strongly supported in our results and is congruent with previous studies based on ultrastructure and multigene phylogenies^7^,^9^. The Ahnfeltiophycidae have naked pit plugs, lacking caps and membranes^33^, and include the Ahnfeltiales and Pihiellales^34^. The latter was not included in this study.

The Rhodymeniophycidae is strongly supported as a monophyletic group with 100% MLB and 1.0 BPP (node ‘6’ in Fig. 1), including 12 orders (out of 13 extant orders^2^) comprising the Acrosymphytales, Bonnemaisoniales, Ceramiales, Gelidiales, Gigartinales, Gracilariales, Halymeniales, Nemastomatales, Peyssonneliales, Plocamiales, Rhodymeniales, and Sebdeniales. All have pit plugs covered by a membrane only; the Gelidiales is an exception in having a thin inner cap under the membrane^21^,^25^. In the Gelidiales, and to a lesser extent the Gracilariales^21^, pit plugs have a striated plug core for which the taxonomic utility has yet to be established. Interordinal relationships of this diverse subclass were not fully resolved in the current study. Nine orders, including Acrosymphytales, Ceramiales, Gelidiales, Gracilariales, Halymeniales, Nemastomatales, Plocamiales, Rhodymeniales, and Sebdeniales form a clade (92% MLB and 0.87 BPP in Supplementary Fig. S1), whereas relationships among the more deeply diverging Bonnemaisoniales, Gigartinales, and Peyssonneliales were largely unresolved (Supplementary Fig. S1). This conflicts with previous multigene phylogenetic analyses that weakly resolved the Ceramiales as an early-diverging group within the Rhodymeniophycidae^7^. However, interrelationships among the orders remain largely unresolved, in particular those subsequent to the divergence of the Peyssonneliales, except for the monophyletic assemblage of the three orders Halymeniales, Rhodymeniales, and Sebdeniales (99% MLB and 0.99 BPP in Supplementary Fig. S1). As indicated in Verbruggen *et al.*^9^, this is one region of the red algal phylogenetic tree that is in need of considerably more investigation.

In Rhodymeniophycidae, all orders were strongly supported as monophyletic groups (98–100% MLB and 1.0 BPP in Supplementary Fig. S1) with the exception of the Gigartinales (61% MLB and 1.0 BPP in Supplementary Fig. S1) and the Plocamiales (52% MLB and 1.0 BPP in Supplementary Fig. S1), which is consistent with published data^7^,^9^,^35^. Once again, relationships in this part of the tree need additional study including improved taxon sampling (e.g., *Hummbrella hydra*, the lone member of the Pseudoanemoniaceae), more sequence data and further exploration of analyses options such as data partitions and evolutionary model selections (see Le Gall *et al.*^7^ and Verbruggen *et al.*^9^). The Ceramiales, the largest florideophycean order, was recovered as monophyletic (98% MLB and 1.0 BPP in Supplementary Fig. S1), and resolved as sister to the Acrosymphytales (‘node 7’ in Fig. 1; 82% MLB, 1.0 BPP in Supplementary Fig. S1). Previous studies^9^,^36^, however, have indicated that the Inkyuleeaceae may not join the remainder of the Ceramiales as a monophyletic group, which when resolved may require further taxonomic revision.

### Divergence time estimation, fossils, and the evolution of florideophyte algae

Divergence time estimation using fossil constraints usually entails a large degree of uncertainty. This is because taxonomic assignment and age determination may be uncertain, and for poorly sampled lineages, the oldest recognized fossils may singificantly postdate the the origin of the group^37^,^38^. For these reasons, we tested the impact of calibration constraints on the estimated divergence times of red algae using the parametric prior distributions available in BEAST. We compared the posterior mean estimates of nodes between the uniform (*uni*) and normal (*nor*) prior distributions. Regression analysis suggests that use of *uni* results in markedly older divergence time estimates than under *nor* (Supplementary Table S1). The correlation coefficient (slope *b*) of estimates (Y) to C7 *nor* (X) showed that *uni* ages (e.g., *b* = 1.4936 for C7 *uni*) were significantly older than *nor* ages (*b* = 0.9794 for C7 *nor*). For instance, the largest differences were found in the node ‘r’ of C7 *uni* age (2,816 Ma 95% High Posterior Density [HPD]: 1,415–5,663 Ma) to C7 *nor* age (1,694 Ma, 95% HPD: 1,484–1,925 Ma), i.e., Δnode ‘r’ mean = 1,122 Ma. The age of node ‘1’ of C7 *uni* (1,661 Ma, 95% HPD: 883–3,443 Ma) was 1.8-fold older than that of C7 *nor* (943 Ma, 95% HPD: 817–1,049 Ma), i.e., Δnode ‘1’ mean = 718 Ma. In the C7 *uni* age, 1,661 Ma for node ‘1’ (the first appearance of the Florideophyceae) was much older than the taxonomically defined oldest eukaryote fossil^12^ (*Bangiomorpha*, node ‘a’). Therefore, we used *nor* estimates for the inferences described below.

Under the *nor* approach, age estimates that removed one constraint ‘b, d–g’ at each time (C6-*b nor*, C6-*d nor*, C6-*e nor*, C6-*f nor*, and C6-*g nor*) and using only the outgroup (C5-*bc nor*), showed a negligible effect (*b* > 0.96). However, removal of constraint ‘a’ (C6*-a nor*, *b* = 0.8491) and using constraint ‘b’ only (C1*b nor*, *b* = 0.8566) led to an underestimation of the divergence time. For example, the age of node ‘r’ in C6-*a nor* (1,362 Ma, ca. 20% younger) and C1*b nor* (1,609 Ma, 5% younger) were younger than that of C7 *nor*. The analyses with red algal fossils only (C2*ab nor*) and the *Bangiomorpha* fossil (C1*a nor*) showed little difference for the age of node ‘r’, i.e., Δnode ‘r’ mean to C7 *nor* were 4 Ma (0.2%) for C2*ab nor* and 20 Ma (1.2%) for C1*a nor*. These results indicate that constraint ‘a’ (early stem group of red algae, *Bangiomorpha*) is more critical for divergence time estimates regarding red algae than the coralline fossils deposited in the Doushantuo formation. Removal of constraint ‘c’ (C6-*c nor*) led to a little overestimation (*b* = 1.1162). For example, the age of ‘node r’ was overestimated in C6-*c nor* age (2,287 Ma). Regardless of distribution priors, the mean age of all nodes using only secondary calibration (C1*c nor* and C1*c uni*) derived from a previous study^16^ resulted in drastcally younger ages for all nodes (Supplementary Table S1). In general, the divergence time estimates and 95% credibility intervals varied among constraint scenarios under both uniform (*uni*) and normal (*nor*) prior distributions. However, florideophycean time estimates from independent analyses overlapped with each other within the 95% HPD.

To test the robustness of the time estimates, we compared results from different speciation tree priors including i) Yule, ii) Birth-Death and iii) Birth-Death Incomplete (see Supplementary Table S2). The date estimations, however, were largely congruent with each other (*b* = 1.0175 with r^2^ = 0.9986 for the Birth-Death and *b* = 1.0163 with r^2^ = 0.9976 for the Birth-Death Incomplete priors to the Yule prior), suggesting highly robust results. For example, the ages of node ‘1’ were 943 Ma, 917 Ma, and 949 Ma with Yule, Birth-Death and Birth-Death Incomplete tree priors, respectively. Based on these comparisons, the normal calibration priors with the Yule speciation process (C7 *nor*) were chosen for the split time estimates for the major florideophycean lineages (Fig. 1).

The divergence time of the Florideophyceae from a common ancestor with the Bangiophyceae was calculated as 943 Ma (95% HPD: 817–1,049 Ma) for node ‘1’ (Fig. 1), a late Mesoproterozoic to early Neoproterozoic^39^ estimate that is consistent with previous results. Lim *et al.*^40^ suggested an origin of red algae in early eukaryotic evolution at 1,300–1,400 Ma, followed by a florideophycean (7 genera) split from *Porphyra* (Bangiophyceae) without any detailed phylogenetic analysis. Yoon *et al.*^10^ estimated the divergence time of florideophytes (ca. 800 Ma) by using the relaxed molecular clock analysis. Although they used multigene data (16 S rRNA, *psaA, psaB, psbA, rbcL*, and *tufA*) as well as multiple fossil constraints including two red algal fossils, only two florideophycean species (*Chondrus* and *Palmaria*) were included in the analysis. Here, we included representatives of almost all florideophycean orders (27 out of 29 orders) with seven fossil constraint data and a seven-gene dataset for the divergence time estimation.

The oldest convincing geological evidence for red algae comes from the erect filamentous microfossils of *Bangiomorpha* preserved by early diagenetic silicification in tidal flat/lagoonal carbonates from Arctic Canada^11^. Radiometric dates on volcanic rock constrain the age of these fossils to be younger than 1,267 ± 2 Ma and older than 723 ± 3 Ma, but an U-Th-Pb whole rock age of 1,092 ± 59 Ma for black shale that underlies the fossiliferous horizon^41^, an unpublished Pb-Pb date on correlative carbonates and arguments from sequence stratigraphy^15^ suggest that the true age lies closer to the lower radiometric boundary. Because the gross morphology of *Bangiomorpha* is similar to that of extant *Bangia* species, it might be recognized as a taxon in crown Bangiophyceae. If correct, this suggests that the divergence of the Bangiophyceae and Florideophyceae occurred prior to 1,200–1,100 Ma, ca. 200–300 Ma earlier than our molecular clock estimate. However, several *Bangiomorpha*-like, simple filamentous species occur among the deeply diverging Compsopogonophyceae (i.e., *Compsopogon, Compopogoniopsis, Erythrotrichia, Rhodochaete*) and Stylonematophyceae (i.e., *Bangiopsis, Purpureofilum, Stylonema*) (see Supplementary Fig. S1). In fact, the original description of *Bangiomorpha* noted that the multicellular holdfast had greater similarity to *Erythrotrichia* than *Bangia*^11^. The diagnostic packet-formation during sexual reproduction in the Bangiales, and as reasonably posited for *Bangiomorpha*, has subsequently been reported in three species of the Compsopogonophyceae indicating that this feature has evolved at least twice within the red algae. It is thus possible that *Bangiomorpha* associates with any one of a number of the deep red algal lineages, possibly even an extinct lineage that evolved characters in parallel to the Bangiophyceae and Compsopogonophyceae. Therefore, it would not be unreasonable to place the *Bangiomorpha* constraint as a stem taxon to the early branching lineages of red algae (node ‘a’ in Fig. 1). Our estimate for the time of the initial red algal divergence casts doubt on the interpretation of budding coccoidal microfossils from the ca. 1,850 Ma Gunflint Formation, Canada, as red algae^42^.

The major divergences within the Florideophyceae (nodes ‘2–5’ in Fig. 1) occurred during the mid–Neoproterozoic to early–Paleozoic eras beginning with the Hildenbrandiophycidae, with an estimated divergence time of 781 (95% HPD: 681–879) Ma (node ‘2’ in Fig. 1). During this time, somewhere between nodes ‘1 and 3’ the so-called triphasic (gonimoblast development on the female gametophyte) life cycle evolved in red algae. Did this happen in the ancestor of all Florideophyceae (between nodes ‘1 and 2’) with subsequent loss in the Hildenbrandiophycideae, or was this subclass ancestrally biphasic with gonimoblast development evolving between nodes ‘2 and 3’? Only through elucidation of the sexual pattern for the Hildenbrandiophycidae will this question be resolved. Whenever it originated, the triphasic pattern is characterized by free-living haploid male and female gametophytes, which produce the gametes. The second phase, a sporophyte called the carposporophyte, involves postfertilization development of diploid gonimoblast filaments on the female gametophyte and produces carpospores. The third phase is a second diploid sporophyte, termed a tetrasporophyte in Florideophyceae because meiosis typically results in four-spored meiosporangia (tetrasporangia) with each haploid tetraspore germinating into a gametophyte. Under the assumption that a successful life history tends to maximize the potential for genetic recombination and genetic diversity from the union of a single pair of gametes, Searles^43^ concluded that selection has favored the evolution of a gonimoblast stage in red algae as compensation for the presumed inefficient fertilization attributed to the absence of motile gametes; however, more recent research has shown that fertilization may not be as inefficient as previously thought (e.g. see Maggs *et al.*^44^).

The oldest known florideophyte fossils occur in Ediacaran rocks from southern China^13^. Half a dozen taxa of thalloid algae preserved in three-dimensional cellular detail by early diagenetic phosphate precipitation reveal features that ally them to florideophyte algae. Whereas several of the preserved populations have been interpreted as early branching florideophytes – perhaps stem groups or early crown representatives of extant taxa – the presence of filamentous “cell fountains,” cortex-medulla differentiation, conceptacles, possible cell fusions, carposporangia, and tetrasporangia (cruciate and stalked) suggest that stem corallines are present in this assemblage as well^13^. Radiometric dates^15^ constrain these fossils to be younger than 632.5 ± 0.5 Ma and older than 551.1 ± 0.7 Ma, and a sequence boundary beneath the fossils might correlate with 580 Ma glaciations in the northern hemisphere. These age constraints are within the 95% credibility intervals of our molecular clock estimates (i.e., estimate without constraint ‘b’) for initial florideophyte diversification. Independent evidence of Ediacaran red algae comes from organic matter containing high abundances of C^27^ steranes^45^ – red algae are unusual among algae for the predominance of C^27^ molecules in their sterol profiles^46^.

The split of the Nemaliophycidae (node ‘3’ in Fig. 1) from the Ahnfeltiophycidae-Corallinophycidae-Rhodymeniophycidae (ACR lineage) occurred 661 (95% HPD: 597–736) Ma. The Corallinophycidae diverged at 579 (95% HPD: 543–617) Ma followed by the split of Ahnfeltiophycidae and Rhodymeniophycidae about 508 (95% HPD: 442–580) Ma (node ‘5’). Diversification within Nemaliophycidae began at 331 (95% HPD: 202–458) Ma (node ‘4’); Nemaliophycidae have cruciate tetrasporangia and mono- and bi-sporangia. Carposporophytes produce spore-bearing gonimoblasts directly from the fertilized carpogonium in this group, and the fertilization nucleus is thus not transferred to separate generative auxiliary cells for production of the gonimoblasts. Cell-to-cell fusions in nemaliophycidaen gonimoblast development are restricted to cells of the carpogonial branch resulting in a secondarily formed carpogonial fusion cell. In this case, as gonimoblast development proceeds, the cytoplasm of the carpogonial branch cells extends and fuses around the cells’ pit plugs with the result that the pit plugs become dislodged and the carpogonial branch cells form open connections with one another, forming a fusion cell. These cell-to-cell fusions do not involve postfertilization connecting cells or connecting filaments. In the Ahnfeltiales (Ahnfeltiophycidae)^33^, as well as the Gelidiales^47^ and Gracilariales^48^ (Rhodymeniophycidae), generative auxiliary cells are not present and the fertilization nucleus remains in the carpogonium, which facultatively fuses with neighbouring vegetative cells following fertilization resulting in another type of carpogonial fusion cell that cuts off gonimoblast initials. In the remainder of the Rhodymeniophycidae, fusion cells may incorporate a generative auxiliary cell, supporting cell, adjacent sterile filaments, and nutritive vegetative gametophyte cells acting as auxiliary cells^49^. In the Rhodogorgonales, auxiliary cells and connecting filaments are absent, and gonimoblast filaments cut off directly from the fertilized carpogonia elongate, with portions of the intercalary gonimoblast cells expanding in size and consecutively initiating secondary gonimoblasts at the point of fusion with terminal cells of specialized vegetative filaments^50^; it is likely that such independent, unique cell-cell fusion mechanisms not involving the production of auxiliary cells was ancestral in corallinophycidaen diversification (asterisk in Fig. 1).

Various types of pre- and post-fertilizational cell-to-cell fusion mechanisms form the basis for classifying the florideophyte red algae and can be found in the remainder of the Rhodymeniophycidae^49^,^51^,^52^, i.e. the Acrosymphytales, Bonnemaisoniales, Ceramiales, Gigartinales, Halymeniales, Nemastomatales, Peyssonneliales, Plocamiales, Rhodymeniales and Sebdeniales. The great diversity in pre- and postfertilization strategies in the Rhodymeniophycidae suggests that the ancestors of these taxa “experimented” on multiple occasions (i.e., at all taxonomic levels) on how to enhance carpospore production from a single fertilization event. Some taxa did it by the direct production of carposporophytes from the fertilized carpogonium, whereas other taxa produced generative auxiliary cells along with a suite of accompanying diploidization strategies. Because many strategies evolved in parallel, along with accompanying reversals, in all of these lineages, considerably better phylogenetic resolution at all taxonomic levels is needed before the evolutionary pathways for these complex postfertilization patterns can be resolved for Rhodymeniophycidae.

The Rhodymeniophycidae is the largest subclass (5,017 spp) of the Florideophycidae, having begun to diversify about 412 (95% HPD: 359–477) Ma (node ‘6’ in Fig. 1). Its members are diverse with respect to many morphological characters in addition to the postfertilization richness discussed above. For example, all three major types of tetrasporangia division are present; viz., zonate, cruciate, and tetrahedral. However, this feature was also gained and lost on numerous occasions, appearing in some, but not all, Acrosymphytales, Ceramiales and Rhodymeniales. Thus, resolving evolutionary pathways of tetrasporangial diversity in this subclass also awaits improved phylogenetic resolution. Lack of interordinal phylogenetic resolution within the Rhodymeniophycidae is a common theme in the literature^7^,^9^,^31^,^36^,^50^,^52^ and remains consistent with our analyses (Supplementary Fig. S1). Indeed, the only relationship consistently resolved is a group^33^,^51^,^52^ including the Halymeniales, Rhodymeniales and Sebdeniales (99% MLB and 0.99 BPP in Supplementary Fig. S1). Clearly better phylogenetic resolution is needed within Rhodymeniophycidae before the evolutionary patterns of the intricate anatomical features characterizing the many species of this subclass can be explored.

The Ceramiales constitutes the most diverse florideophyte order (2,654 spp) with a divergence time estimated as 335 (95% HPD: 284–395) Ma (node ‘7’ in Fig. 1). The Ceramiales is distinguished from other florideophycean species by the formation of auxiliary cells after fertilization. Our relaxed clock analysis suggests that this unifying feature evolved during the Devonian (419–359 Ma) to Carboniferous (359–299 Ma) periods^39^ of the Paleozoic Era.

With the exception of the *Conchocelis* stage of *Porphyra*-like algae, preserved as endoliths in Paleozoic carbonates^53^,^54^, red algae are represented in Phanerozoic rocks largely as calcareous skeletons. Several extant florideophyte clades are known to precipitate CaCO_3_: as aragonite in a few members of the Peyssonneliales and Nemaliales, and as calcite in the Corallinales (Ca(Mg)CO_3_), Sporolithales, Hapalidiales, and Rhodogorgonales. With some uncertainty, representatives of the first two groups have been reported from Carboniferous and Permian (>350 Ma) carbonates^55^,^56^, consistent with our molecular clock inference of major florideophyte diversification during the late Paleozoic Era. Remnants of stem group corallines that contain features of biocalcification, partitions, calcified sporangial compartments and trichocytes, occur as well, and can be traced back to the Ordovician Period (485–445 Ma)^57^,^58^, after the emergence of non-calcified corallines preserved in the Doushantuo rocks (635–551 Ma). The evolution of skeletal biomineralization within this clade occurred within the context of an ecosystem-wide increase in carbonate skeletonization that characterizes the Ordovician marine radiation^59^, reflecting an increase in predation pressure, a change in seawater chemistry, or both^60^.

In contrast, crown group diversification of coralline algae appears to be restricted to mid-Mesozoic and younger oceans, as indicated by both fossils and molecular clock estimates^16^,^17^. This places coralline diversification within the context of the Mesozoic marine revolution during which many skeleton-forming clades evolved protective responses to the radiation of shell-crushing predators^61^,^62^. We note however that the single-gene based molecular clock analysis of Aguirre *et al.*^16^, which does not take into account stem group corallines in Ediacaran to Ordovician rocks^13^,^14^,^15^,^54^,^57^,^58^, yields estimates of family level diversification within the Corallinales that are about two times younger than our estimates. This approach also yields an age for the divergence of corallines from nemalialeans (338.26 Ma) far younger than our estimate (579 Ma). If we accept that the Doushantuo Formation contains stem group corallines, then the estimate of Aguirre *et al.*^16^ for the coralline-nemalialean divergence must be too young. One might relax the interpretation that the Doushantuo fossils are stem group corallines, but any clock that accepts them as at least stem group florideophytes is unlikely to yield a Carboniferous date for the nemalialean-coralline divergence. Brooke and Riding^56^ interpret Ordovician fossils as corallines and perhaps even stem group Sporolithaceae; this would also require a divergence within the Corallinales older than that estimated by Aguirre *et al.*^16^. For now, it may be most judicious to note the difference in estimates governed by different calibration strategies, and look to continuing improved sampling of living florideophytes as well as better paleontological constraints, in particular for Paleozoic and older fossils.

Furthermore, when we compared time estimates with/without the two constraints of Aguirre *et al.* (‘c1’ for the split of the Sporolithales of 133 Ma, and ‘c2’ for the split of Corallinaceae and Hapalidiaceae of 117 Ma based on the Cenozoic corallines^17^), all divergence times were within the 95% credibility interval (see Supplementary Table S1 and Fig. S2). It may be that the crown group divergences within the Corallinaceae and Sporolithaceae is accurately captured by the fossil record^17^ but that divergence between the two clades was much earlier. Our molecular clock based on seven-genes with broad taxon sampling is consistent with fossils in suggesting that the coralline lineage diverged from other rhodophytes long before the crown group radiation of corallines. This, in turn, is consistent with other evidence that shows the time interval between total group divergence and crown group diversification can be long in eukaryotic clades^63^.

Few molecular clock analyses have estimated the divergence times within the red algae. In the analysis of Parfrey *et al.*^64^, which included 88 taxa (4 of them rhodophytes) and 15 genes, divergence estimates depended on molecular model and choice of paleontological constraints. In general however, time estimates for radiation within the Rhodymeniophycidae, the Bangiophyceae-Florideophyceae split, and the initial divergence of red algae are similar to or slightly younger than those reported here. More broadly, both fossils^65^ and several recent molecular clock analyses^66^,^67^ suggest a mid-Proterozoic origin of photosynthetic eukaryotes but Neoproterozoic and later diversification of taxa within the major clades of Archaeplastida. In contrast, the molecular clock estimates of Berney and Pawlowski^67^ suggest that red and green algae diverged only about 900 million years ago.

## Conclusion

We studied the major diversification events within red algae using a multigene dataset (concatenated genes of nuclear EF2, LSU and SSU rRNAs, plastid encoded *psa*A, *psb*A, and *rbc*L, and mitochondrial *cox*1). Our ML phylogeny supports a sister group relationship between the Bangiophyceae and Florideophyceae, and resolves relationships among the five subclasses of the Florideophyceae. The multigene relaxed clock estimation using multiple fossil constraints suggests that florideophytes arose near the beginning of the Neoproterozoic Era. The major evolutionary divergences within the class occurred in mid-Neoproterozoic to early Paleozoic oceans, beginning with the split of the Hildenbrandiophycidae followed by the appearance of the Nemaliophycidae, Corallinophycidae, Ahnfeltiophycidae and Rhodymeniophycidae. Radiation of the rhodymeniophycidaen algae is thought to have occurred during the mid Paleozoic Era.

These major divergences were accompanied by evolutionary innovations in the carposporophyte stage, maximizing spore production from each fertilization event. The Nemaliophycidae and Ahnfeltiophycidae and Corallinophycidae (in part) did not evolve generative auxiliary cells and the site of fertilization and diploidization in these taxa thus remain restricted to the carpogonium. Nonetheless, in most Rhodymeniophycidae and Corallinophycidae (in part), these two processes (likely independently) became decoupled with this division of labor resulting in the great diversity of carposporophyte types that characterize each of the subclasses. It is precisely the sequence of events starting with the establishment of the female reproductive system in relation to vegetative growth, and leading to the postfertilization carposporophyte that has traditionally formed the basis of red algal classification.

Our research provides the first comprehensive estimation of divergence dates within the Florideophyceae using molecular and fossil data. Although these results are clearly working hypotheses, we are buoyed by the observation that there was considerable congruence between the current results with both previous time estimates and the fossil record. Future studies that incorporate additional red algal diversity should be used to test the ideas put forth in our study.

## Methods

### Taxon sampling and sequencing strategy

To establish a well-resolved phylogeny for the red algae, we selected 91 red algal taxa representing 34 orders, 27 of them florideophycean and seven non-florideophycean^6^,^7^,^68^. We generated 180 new sequences for *rbc*L (n = 49), *psa*A (61), *psb*A (58), EF2 (2), SSU (1), LSU (2), and *cox1* (7) genes from florideophycean taxa to reduce missing data in the multigene alignment. Publicly available sequences of red algae, eight green algal representatives (including land plants), and three cyanobacterial species were downloaded from GenBank (Supplementary Table S3). The cyanobacteria *Nostoc* sp. PPC 7120, *Synechocystis* sp. PCC 6803, and *Thermosynechococcus elongatus* BP-1 were used as an outgroup to root the tree.

Genomic DNA was extracted from approximately 5 mg of algal biomass that had been pulverized in liquid nitrogen. The DNeasy Plant Mini Kit (Qiagen GmbH, Hilden, Germany) or Invisorb Spin Plant Mini Kit (Invitek, Berlin-Buch, Germany) was used for DNA extraction following the manufacturers’ instructions. PCR and sequencing reactions were conducted using specific primers for each of three plastid genes^69^: psaA130F, psaA971F, psaA1110R, psaA1530F, psaA1760R, and psaA-3 for *psa*A; psbA-F, psbA-R1, psbA-500 F, psbA-600 R, and psbA-R2 for *psb*A; and F7, R753, F645, and RrbcS start for *rbc*L. PCR amplification was performed in a total volume of 25 μl that contained 0.5 U *TaKaRa Ex Taq*^TM^ DNA polymerase (Takara Shuzo, Shiga, Japan), 2.5 mM of each dNTP, 2.5 ul of the 10X *Ex Taq*^TM^ Buffer (Mg^2+^ free), 2 mM MgCl_2_, 10 pmol of each primer, and 1–10 ng template DNA. The reaction was carried out with an initial denaturation at 94 °C for 10 min, followed by 35 cycles of amplification (denaturation at 94 °C for 30 sec, annealing at 50 °C for 30 sec, and extension at 72 °C for 2 min), with a final extension at 72 °C for 10 min. The PCR products were purified using a High Pure PCR Product Purification Kit (Roche Diagnostics GmbH, Mannheim, Germany), in accordance with the manufacturers’ instructions. The sequences of the forward and reverse strands were determined for all taxa using commercial sequencing services. The electropherogram output for each specimen was edited using the program Chromas Lite v.2.1.1. (http://www.technelysium.com.au/chromas.html).

### Alignment and phylogeny

Alignments were generated manually using Se-Al v.2.0a11 (http://tree.bio.ed.ac.uk/software/seal/). Because *rbc*L sequences of green algae and land plants are derived from the cyanobacterial primary endosymbiont rather than from a proteobacterium, as in red algae, they were coded as missing data. We used a mixed model of amino acid and DNA sequences for phylogenetic analysis. Translated amino acid sequences for protein-coding genes were used to reduce the possibly misleading effects of nucleotide bias or mutational saturation in our data sets. In addition, only conserved regions of the rDNA alignment were used for phylogenetic analysis. The final dataset contained 102 taxa, comprising a total of 6,966 characters, including 2,053 amino acid positions (500 *psa*A, 301 *psb*A, 463 *rbc*L, 568 EF-2, and 221 *cox*1) and 4,913 DNA positions (3,070 LSU and 1,843 SSU). The final alignment is available upon request from HSY and the Supplementary Information of the journal website at http://www.nature.com/naturecommunications/.

Appropriate evolutionary models were selected separately for each protein and DNA alignment. For the amino acid dataset, the best-fit model was chosen using ModelGenerator v.0.85 (http://bioinf.nuim.ie/modelgenerator/) and preliminary Bayesian analyses using MrBayes v.3.2^70^. The LG model was selected by ModelGenerator as a best fit from available amino acid models under the both AIC (Akaike information criterion) and BIC (Bayesian information criterion). We used the LG model for Maximum Likelihood (ML) analyses using RAxML v.8.1^71^. Because MrBayes does not support the LG model, the CPREV substitution model was selected for the Bayesian inference (BI). For the DNA alignment, the GTR substitution model was used.

ML and bootstrap analyses of protein + DNA combined data were conducted under the LG and GTR with independent rate heterogeneity (LG + F + G and GTR + G mixed model) using RAxML. We used 100 independent tree inferences using the default option of which automatically optimized SPR rearrangement and 25 distinct rate categories for this program to identify the best tree. Bootstrap values were calculated with 1,000 replicates using the same substitution model.

Bayesian posterior probabilities from the combined data were estimated under the CPREV + G and GTR + G mixed model using MrBayes. Two independent Metropolis-coupled Markov chain Monte Carlo (MCMCMC) runs with 20 million generations with four chains were run simultaneously. Every 200^th^ generation tree was sampled and compared to determine the burn-in point. Based on the average standard deviation of split frequencies between two independent runs, 5,000,000 generations were determined to appropriate burn-in point. The remaining 150,002 trees from two runs sampled from the putative stationary distribution were used to infer Bayesian posterior probabilities (BPP) at the nodes. The ML and Bayesian tree topologies were congruent with each other.

### Molecular clock analysis

The ML tree from RAxML was used to estimate divergence times. The hypothesis of rate constancy among taxa was tested by comparing likelihoods using the likelihood ratio test, given the best ML tree topology with and without the constraint of a molecular clock. As expected from the heterogenous branch lengths, a clock-like evolution of substitution rates was rejected for our data set (data not shown; see also Yoon *et al.*^10^). Thus, the multigene relaxed clock methods were used to estimate divergence times. The Bayesian relaxed dating was conducted using the BEAST v.1.8.1 software package^20^. This method accommodates rate variation both among lineages and among genes. Branch lengths were estimated using only combined protein data under a LG model. We used combined data (DNA + protein; two partitions) with the seperate GTR + G and LG + F + G models for each part. Topologies and branch lengths were linked for partitions and other parameters were unlinked, i.e., substitution matrixes, base or amino acid frequencies, and gamma parameters. The Yule process prior for speciation and uncorrelated lognormal relaxed clock models were used. The prior constraint time of selected nodes were set with upper and lower limits by using normal distribution of mean and standard deviation. The MCMC analyses were run for 20 million generations with sampling every 1,000^th^ generation. Convergence of the MCMC algorithm was assessed by plotting of likelihood value. Initial 5 million generations were discarded as burn-in. In addition, the Birth-Death and Birth-Death Incomplete speciation priors were used to test the robustness of the time estimation (see Supplementary Table S2). TreeAnnotator (part of BEAST package) was used to visualize the maximum clade credibility phylogeny and time estimation.

The following seven constraints were used for divergence time estimation (see Supplementary Fig. S1). The first node ‘a’ was constrained at 1,174–1,222 (1,198 ± 12) Ma based on a Pb-Pb date for carbonates correlative with well-preserved fossils of the multicellular filamentous red alga *Bangiomorpha*^11^,^12^. Because there are simple filamentous species within the Compsopogonophyceae and Stylonematophyceae (i.e., *Erythrotrichia, Compsopogon*, *Rhodochaete*, *Bangiopsis*, and *Stylonema*), we used node ‘a’ for the constraint point for the *Bangiomorpha* fossil data, instead of the node ‘1’ ancestral to the Bangiophyceae and Florideophyceae (see Supplementary Fig. S2). Coralline algae (Corallinophycidae) provide an excellent context to stratigraphy^13^,^14^,^15^ and molecular clock analysis because of their calified cell wall structure^16^,^17^. We constrained node ‘b’ to a date of 635–551 (593 ± 21) Ma based on an early stem group of corallinalean red algae from the Doushantuo Formation, China^13^,^14^,^15^. The Doushantuo Formation contains the stem group of corallines, i.e., *Thallophyca* and *Paramecia*. Light microscopy and SEM data revealed fossilized features of the coralline algae such as complex pseudoparenchymatous thalli, specialized reproductive structures (carposporophytes, tetrasporangia and tetraspore-like cells) and cell to cell fusion^14^. The U-Pb zircon dates of volcanic ash beds within the Doushantuo Formation, animal fossils, and synchronous deglaciation records support the age of the formation^15^. Other coralline data from Aguirre *et al.*^16^,^17^ were available for calibration points, i.e., 130–136 (133 ± 1.5) Ma for node ‘c1’ for the Sporolithales split, and 114–120 (117 ± 1.5) Ma for ‘c2’ for the split of Corallinales and Hapalidiales. The four nodes marked ‘d–g’ were designated constraints of the Viridiplantae (green algae and plants) used in a recent study^18^, including three fossil constraints, i.e., 471–480 (475.5 ± 2.25) Ma for the age of land plants (node ‘d’), 401–422 (411.5 ± 5.25) Ma for the age of the euphyllophytes (node ‘e’), 313–351 (332 ± 9.5) Ma for the age of the seed plants (node ‘f’), and 138–163 (150.5 ± 6.25) Ma for the split of Eudicotyledoneae (node ‘g’).

To test how the calibrations affected the age estimation of red algae, we devised 14 distinct calibration scenarios based on the constraint category^37^ (Supplementary Table S1). The *Bangiomorpha* fossil (constraint ‘a’) and green plant lineage (constraints ‘d–g’) were considerd “outgroup” constraints. Coralline fossils of the Doushantuo formation (constraint ‘b’) and estimated times of coralline classes (constraints ‘c1 and c2’) were consided “ingroup” constraints of the Florideophyceae. The constraint ‘b’ was “safe” because it showed high level of taxonomic resolution and accurate age^14^,^15^. However, constraints ‘c1 and c2’ were considered “risky” because these were “secondary” calibrations derived from a previous estimation^16^,^17^. In the first calibration scenario, we used all seven age constraints (C7) for age estimation at the nodes. In the second, we used six constraints excluding one constraint (C6-*i*, *i* = a–g) in order to evaluate the impact of each constraint. The constraint ‘c’ is only a “secondary” constraint in the present study, in order to provide an empirical test for the impact of the probability range in the normal prior distribution (mean and S.D.). We also applied additional analyses with two and ten times the standard deviation (S.D.), i.e., C6-*c 2* *SD* and C6-*c 10* *SD*. In the third calibration scenario we compared two analyses with “outgroup” only (C5–*bc*) and “green” lineage only (C4-*abc*). In the fourth, we used a red algal constraint only, i.e., C2*ab* (constrains ‘a’ and ‘b’) and C1*a*, C1*b*, and C1*c* (constraint ‘a’, ‘b’, and ‘c’, respectively).

The parametric prior distributions were compared using Bayesian inference implemented in the BEAST package. In the uniform prior distribution, all calibrations were specified with minimum boundary and arbitrarily large maximum boundary (1.0e100). In the normal prior distribution, all calibrations were specified with minimum and maximum boundaries described above. We compared the ages of all estimates (Y; dependent variable) and those under the C7 scenario with normal prior distribution (C7 *nor*, X; independent variable) by regression analysis (Y = *a* + *b*X; Supplementary Table S1).

## Additional Information

**How to cite this article**: Yang, E. C. *et al.* Divergence time estimates and the evolution of major lineages in the florideophyte red algae. *Sci. Rep.* **6**, 21361; doi: 10.1038/srep21361 (2016).

## Supplementary Material

Supplementary Information

Supplementary Dataset 1
