# Supplementary material for: Divergence time estimates and the evolution of major lineages in the florideophyte red algae
Source: Sci Rep. 2016 Feb 19;6:21361. doi: 10.1038/srep21361 (PMC4759575; doi:10.1038/srep21361)
Supplement: Supplementary Information [file srep21361-s1.pdf]

Supplementary Information

Divergence time estimates and evolution of major lineages in the florideophyte red algae

Eun Chan Yang[1,2], Sung Min Boo[3], Debashish Bhattacharya[4], Gary W Saunders[5], Andrew H Knoll[6], Suzanne Fredericq[7], Louis Graf[8], & Hwan Su Yoon[8]*

[1]Marine Ecosystem Research Division, Korea Institute of Ocean Science & Technology, Ansan 15627, Korea

[2]Department of Marine Biology, Korea University of Science and Technology, Daejeon 34113, Korea

[3]Department of Biology, Chungnam National University, Daejeon 305-764, Korea

[4]Department of Ecology, Evolution and Natural Resources, Rutgers University, New Brunswick, NJ 08901, USA

[5]Department of Biology, University of New Brunswick, Fredericton, NB E3B 5A3 Canada

[6]Department of Organismic and Evolutionary Biology, Harvard University, Cambridge, MA 02138, USA

[7]Department of Biology, University of Louisiana at Lafayette, Lafayette, LA 70504-2451, USA

[8]Department of Biological Sciences, Sungkyunkwan University, Suwon 16419, Korea

List of files

Supplementary Figure S1. Phylogenetic relationships of the Florideophyceae. Tree of the Florideophyceae with other red algal classes and green plants inferred by a likelihood analysis using the concatenated EF2, *cox*1, *psa*A, *psb*A, *rbc*L (protein), and LSU, SSU (DNA) supermatrix under the LG+F+G and GTR+G mixed model. Maximum likelihood bootstrap (MLB) support value and Bayesian posterior probabilities (BPP) are shown near the branches. Red algal systematic ranks (order, subclass, and class) are indicated by the taxon name. The characters 'a-g' in dark circle indicates that fossil or molecular based constraint points for relaxed molecular clock estimation. Numbers '1-7' in dark circle indicates major divergence points in the Florideophyceae.

Supplementary Figure S2. The best phylogeny of red algae with selected 31 nodes including seven constraints.

Supplementary Table S1. The constraint scenarios, age estimates (in Ba) for selected nodes, and regression analysis results with the normal and uniform distribution priors.

Supplementary Table S2. Time estimations comparison resulted in the BEAST analyses under different priors, i) Yule Process, ii) Birth-Death and iii) Birth-Death Incomplete process.

Supplementary Table S3. Species examined in the present study. The sequence determined in this study are shown in bold text.

Supplementary Data1. Seven gene combined alignment. The data set used in the present study. Nexus format alignment included partition and the best tree topology which have been inferred from the maximum likelihood search and used in molecular clock estimations.

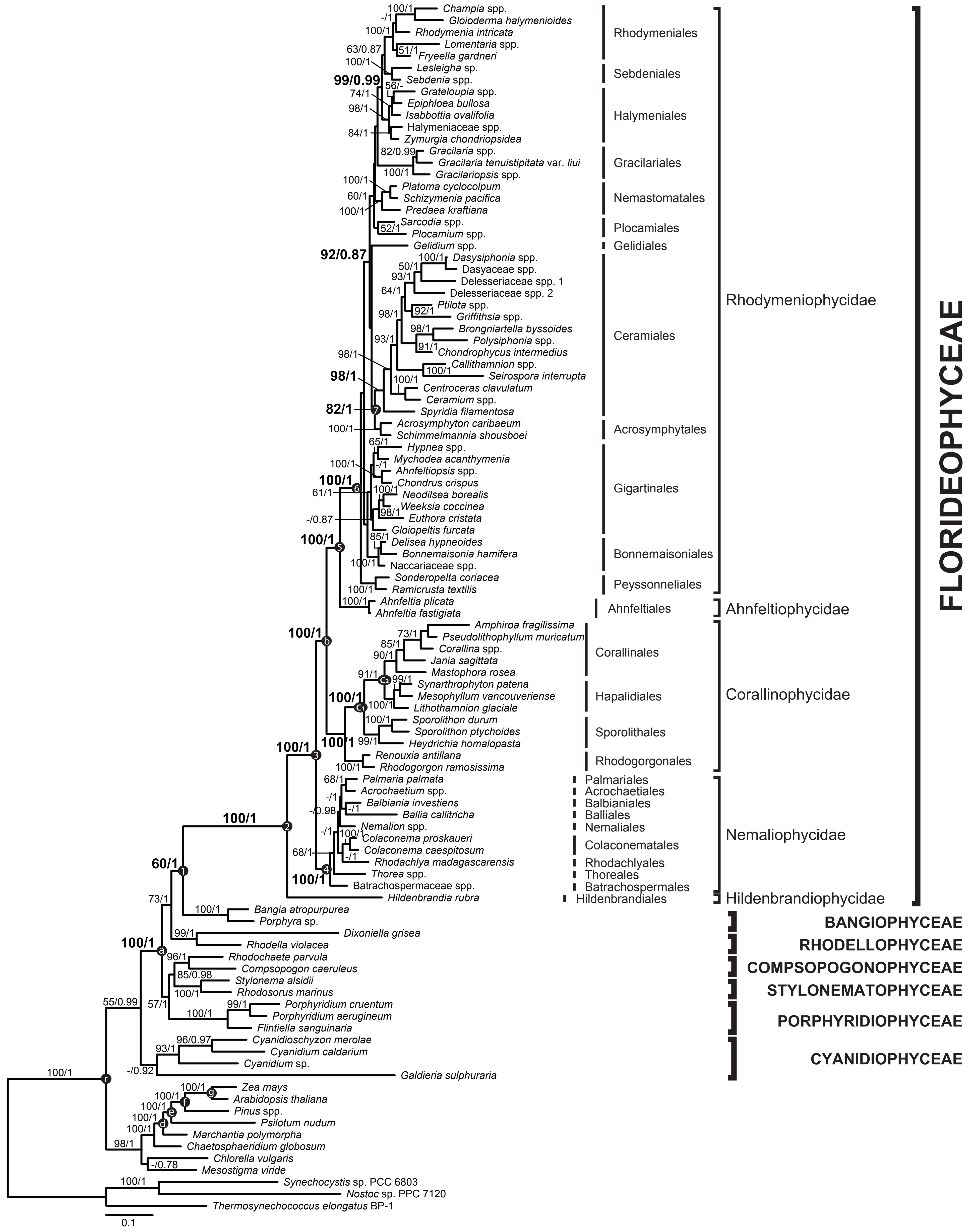
Champia spp.
Gloioderma halymenioides
Rhodymenia intricata
Lomentaria spp.
Fryeella gardneri
Lesleigha sp.
Sebdenia spp.
Grateloupia spp.
Epiphloea bullosa
Isabbottia ovalifolia
Halymeniaceae spp.
Zymurgia chondriopsidea
Gracilaria spp.
Gracilaria tenuistipitata var. liui
Gracilariopsis spp.
Platoma cyclocolpum
Schizymenia pacifica
Predaea kraftiana
Sarcodia spp.
Plocamium spp.
Gelidium spp.
Dasysiphonia spp.
Dasyaceae spp.
Delesseriaceae spp. 1
Delesseriaceae spp. 2
Ptilota spp.
Griffithsia spp.
Brongniartella byssoides
Polysiphonia spp.
Chondrophycus intermedius
Callithamnion spp.
Seirospora interrupta
Centroceras clavulatum
Ceramium spp.
Spyridia filamentosa
Acrosymphyton caribaeum
Schimmelmannia shousboei
Hypnea spp.
Mychodea acanthymenia
Ahnfeltiopsis spp.
Chondrus crispus
Neodilsea borealis
Weeksia coccinea
Euthora cristata
Gloiopeltis furcata
Delisea hypneoides
Bonnemaisonia hamifera
Naccariaceae spp.
Sonderopelta coriacea
Ramicrusta textilis
Ahnfeltia plicata
Ahnfeltia fastigiata
Amphiroa fragilissima
Pseudolithophyllum muricatum
Corallina spp.
Jania sagittata
Mastophora rosea
Synarthrophyton patena
Mesophyllum vancouveriense
Lithothamnion glaciale
Sporolithon durum
Sporolithon ptychoides
Heydrichia homalopasta
Renouxia antillana
Rhodogorgon ramosissima
Palmaria palmata
Acrochaetium spp.
Balbiania investiens
Ballia callitricha
Nemalion spp.
Colaconema proskaueri
Colaconema caespitosum
Rhodachlya madagascarensis
Thorea spp.
Batrachospermaceae spp.
Hildenbrandia rubra
Bangia atropurpurea
Porphyra sp.
Dixoniella grisea
Rhodella violacea
Rhodochaete parvula
Compsopogon caeruleus
Stylonema alsidii
Rhodosorus marinus
Porphyridium cruentum
Porphyridium aerugineum
Flintiella sanguinaria
Cyanidioschyzon merolae
Cyanidium caldarium
Cyanidium sp.
Galdieria sulphuraria
Zea mays
Arabidopsis thaliana
Pinus spp.
Psilotum nudum
Marchantia polymorpha
Chaetosphaeridium globosum
Chlorella vulgaris
Mesostigma viride
Synechocystis sp. PCC 6803
Nostoc sp. PPC 7120
Thermosynechococcus elongatus BP-1
0.1
Rhodymeniales
Sebdeniales
Halymeniales
Gracilariales
Nemastomatales
Plocamiales
Gelidiales
Ceramiales
Acrosymphytales
Gigartinales
Bonnemaisoniales
Peyssonneliales
Ahnfeltiales
Corallinales
Hapalidiales
Sporolithales
Rhodogorgonales
Palmariales
Acrochaetiales
Balbianiales
Balliales
Nemaliales
Colaconematales
Rhodachlyales
Thoreales
Batrachospermales
Hildenbrandiales
Rhodymeniophycidae
Ahnfeltiophycidae
Corallinophycidae
Nemaliophycidae
Hildenbrandiophycidae
FLORIDEOPHYCEAE
BANGIOPHYCEAE
RHODELLOPHYCEAE
COMPSOPOGONOPHYCEAE
STYLONEMATOPHYCEAE
PORPHYRIDIOPHYCEAE
CYANIDIOPHYCEAE

Figure S1

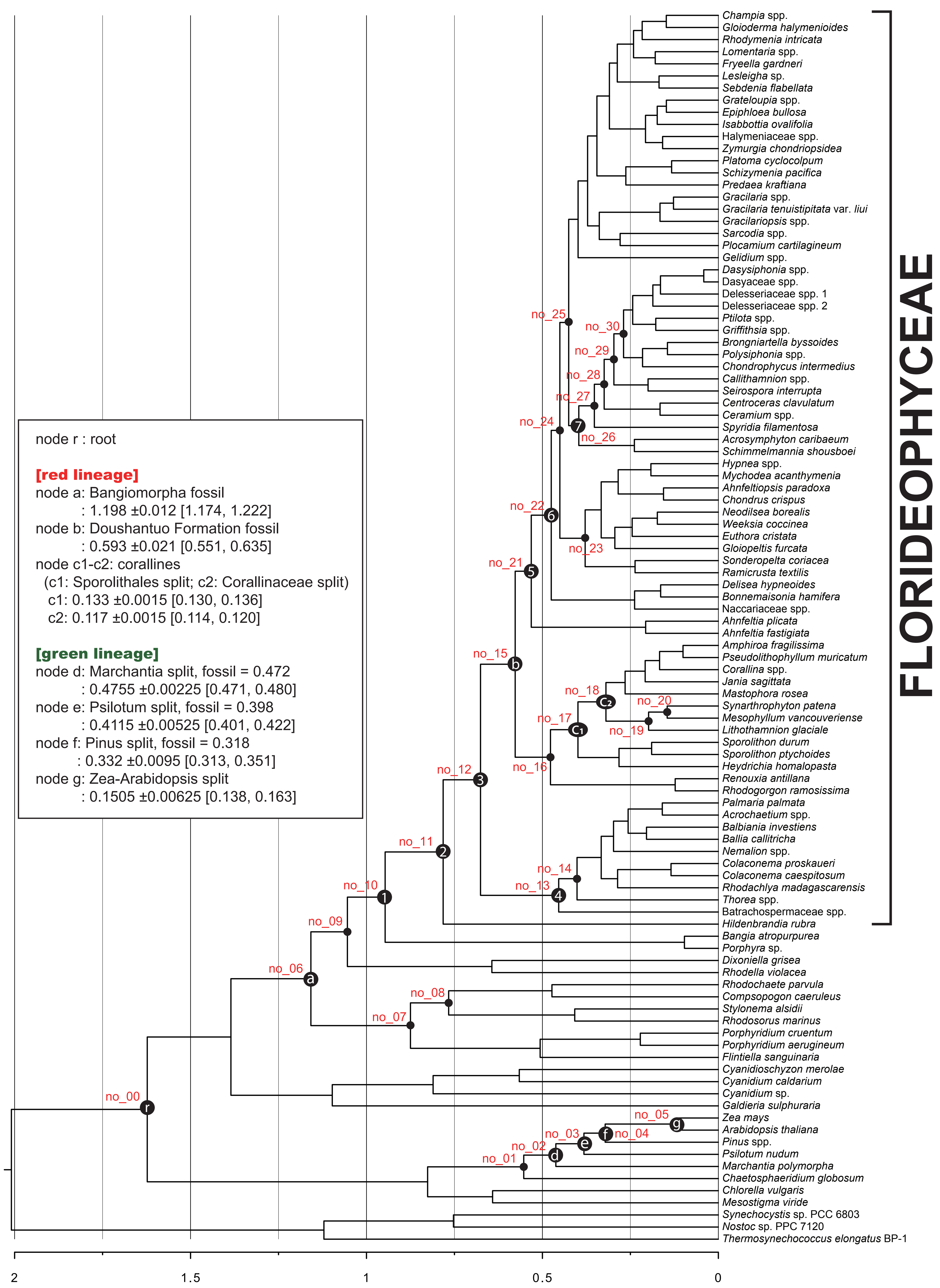

node r : root
[red lineage]
node a: Bangiomorpha fossil
: 1.198 ±0.012 [1.174, 1.222]
node b: Doushantuo Formation fossil
: 0.593 ±0.021 [0.551, 0.635]
node c1-c2: corallines
(c1: Sporolithales split; c2: Corallinaceae split)
c1: 0.133 ±0.0015 [0.130, 0.136]
c2: 0.117 ±0.0015 [0.114, 0.120]
[green lineage]
node d: Marchantia split, fossil = 0.472
: 0.4755 ±0.00225 [0.471, 0.480]
node e: Psilotum split, fossil = 0.398
: 0.4115 ±0.00525 [0.401, 0.422]
node f: Pinus split, fossil = 0.318
: 0.332 ±0.0095 [0.313, 0.351]
node g: Zea-Arabidopsis split
: 0.1505 ±0.00625 [0.138, 0.163]
FLORIDEOPHYCEAE
Champia spp.
Gloioderma halymenioides
Rhodymenia intricata
Lomentaria spp.
Fryeella gardneri
Lesleigha sp.
Sebdenia flabellata
Grateloupia spp.
Epiphloea bullosa
Isabbottia ovalifolia
Halymeniaceae spp.
Zymurgia chondriopsidea
Platoma cyclocolpum
Schizymenia pacifica
Predaea kraftiana
Gracilaria spp.
Gracilaria tenuistipitata var. liui
Gracilariopsis spp.
Sarcodia spp.
Plocamium cartilagineum
Gelidium spp.
Dasysiphonia spp.
Dasyaceae spp.
Delesseriaceae spp. 1
Delesseriaceae spp. 2
Ptilota spp.
Griffithsia spp.
Brongniartella byssoides
Polysiphonia spp.
Chondrophycus intermedius
Callithamnion spp.
Seirospora interrupta
Centroceras clavulatum
Ceramium spp.
Spyridia filamentosa
Acrosymphyton caribaeum
Schimmelmannia shousboei
Hypnea spp.
Mychodea acanthymenia
Ahnfeltiopsis paradoxa
Chondrus crispus
Neodilsea borealis
Weeksia coccinea
Euthora cristata
Gloiopeltis furcata
Sonderopelta coriacea
Ramicrusta textilis
Delisea hypneoides
Bonnemaisonia hamifera
Naccariaceae spp.
Ahnfeltia plicata
Ahnfeltia fastigiata
Amphiroa fragilissima
Pseudolithophyllum muricatum
Corallina spp.
Jania sagittata
Mastophora rosea
Synarthrophyton patena
Mesophyllum vancouveriense
Lithothamnion glaciale
Sporolithon durum
Sporolithon ptychoides
Heydrichia homalopasta
Renouxia antillana
Rhodogorgon ramosissima
Palmaria palmata
Acrochaetium spp.
Balbiania investiens
Ballia callitricha
Nemalion spp.
Colaconema proskaueri
Colaconema caespitosum
Rhodachlya madagascarensis
Thorea spp.
Batrachospermaceae spp.
Hildenbrandia rubra
Bangia atropurpurea
Porphyra sp.
Dixoniella grisea
Rhodella violacea
Rhodochaete parvula
Compsopogon caeruleus
Stylonema alsidii
Rhodosorus marinus
Porphyridium cruentum
Porphyridium aerugineum
Flintiella sanguinaria
Cyanidioschyzon merolae
Cyanidium caldarium
Cyanidium sp.
Galdieria sulphuraria
Zea mays
Arabidopsis thaliana
Pinus spp.
Psilotum nudum
Marchantia polymorpha
Chaetosphaeridium globosum
Chlorella vulgaris
Mesostigma viride
Synechocystis sp. PCC 6803
Nostoc sp. PPC 7120
Thermosynechococcus elongatus BP-1
no_00 r
no_01
no_02 d
no_03 e
no_04 f
no_05 g
no_06 a
no_07
no_08
no_09
no_10 1
no_11 2
no_12 3
no_13 4
no_14
no_15 b
no_16
no_17 c1
no_18 c2
no_19
no_20
no_21 5
no_22 6
no_23
no_24 7
no_25
no_26
no_27
no_28
no_29
no_30
2
1.5
1
0.5
0

Figure S2

Table S1. The constraint scenarios, age estimates (in Ba) for selected nodes, and regression analysis results with the normal and uniform distribution priors.

a.	Bangiomorpha fossil	red algae	out
b.	Doushantuo Formation fossil	red algae	in
c.	Corallines estimation	red algae	in
d.	Land plants age	green algae	out
e.	Euphyllophytes split	green algae	out
f.	Seed plants split	green algae	out
g.	Eudicotyledoneae split	green algae	out

Prior for constraint: Normal distribution (*nor*)

Node id	C7	C7 (2SD)	C7 (10SD)	C6 -a	C6 -b	C6 -c	C6 -c (2SD)	C6 -c (10SD)	C6 -d	C6 -e	C6 -f	C6 -g	C5 -bc	C4 -abc	C2 ab	C1 a	C1 b	C1 c
a	+	+	+		+	+	+	+	+	+	+	+	+		+	+		
b	+	+	+	+		+	+	+	+	+	+	+			+		+	
c1, c2	+	+	+	+	+				+	+	+	+						+
d	+	+	+	+	+	+	+	+		+	+	+	+	+				
e	+	+	+	+	+	+	+	+	+		+	+	+	+				
f	+	+	+	+	+	+	+	+	+	+		+	+	+				
g	+	+	+	+	+	+	+	+	+	+	+		+	+				
note:		x2 S.D.	x10 S.D.				x2 S.D.	x10 S.D.					out only	green only	red fossils			
**r — node_00**	1.6936	1.7008	1.2307	1.3618	1.6740	2.2869	2.0056	1.3448	1.7369	1.6802	1.6761	1.7618	1.9657	n.a.	1.6898	1.7144	1.6089	0.5221
node_01	0.4341	0.4351	0.3794	0.4369	0.4376	0.4309	0.4297	0.4036	0.4780	0.4219	0.4351	0.4355	0.4326	0.4322	0.5818	0.6008	0.5605	0.1809
**d — node_02**	0.4093	0.4050	0.3357	0.4100	0.4099	0.4090	0.4032	0.3567	0.4118	0.3898	0.4086	0.4097	0.4091	0.4087	0.4907	0.5019	0.4681	0.1493
**e — node_03**	0.3611	0.3546	0.2730	0.3659	0.3571	0.3647	0.3545	0.2931	0.3648	0.3545	0.3273	0.3642	0.3624	0.3634	0.3989	0.4032	0.3756	0.1205
**f — node_04**	0.3282	0.3147	0.2290	0.3279	0.3209	0.3262	0.3135	0.2445	0.3283	0.3242	0.2674	0.3270	0.3265	0.3258	0.3324	0.3378	0.3120	0.1026
**g — node_05**	0.1503	0.1505	0.1072	0.1502	0.1504	0.1501	0.1510	0.1327	0.1502	0.1505	0.1499	0.1439	0.1504	0.1507	0.1593	0.1806	0.1556	0.0544
**a — node_06**	1.1943	1.1799	0.7443	1.1945	1.1940	1.1958	1.1894	0.9749	1.1952	1.1943	1.1948	1.1943	1.1956	1.2255	1.1960	1.1975	1.1271	0.3565
node_07	0.6938	0.6054	0.4694	0.6033	0.6016	0.8220	0.8456	0.7231	0.6502	0.6255	0.5926	0.7094	0.8261	0.9713	0.8716	0.9303	0.8489	0.2764
node_08	0.5983	0.5171	0.4092	0.5140	0.5226	0.7182	0.7355	0.6345	0.5610	0.5352	0.5111	0.6114	0.7229	0.8479	0.7655	0.8095	0.7431	0.2420
node_09	1.0596	1.0313	0.6751	0.9006	1.0302	1.0994	1.0781	0.8962	1.0757	1.0454	1.0840	1.0642	1.0734	1.1431	1.0951	1.1076	1.0427	0.3288
(1) — node_10	0.9432	0.9097	0.6063	0.8541	0.9105	0.9973	0.9710	0.8107	0.9717	0.9342	0.9888	0.9557	0.9575	1.0983	0.9823	1.0055	0.9495	0.2997
(2) — node_11	0.7805	0.7231	0.4416	0.7235	0.6734	0.7843	0.7832	0.6548	0.7504	0.7607	0.7591	0.7506	0.7751	1.0163	0.7979	0.8570	0.7629	0.2525
(3) — node_12	0.6608	0.6088	0.3777	0.6317	0.5559	0.6803	0.6798	0.5754	0.6479	0.6423	0.6495	0.6542	0.6623	0.8798	0.6940	0.7520	0.6724	0.2227
(4) — node_13	0.3312	0.3262	0.2440	0.3178	0.3268	0.4355	0.4290	0.3747	0.3383	0.3464	0.3290	0.3707	0.4006	0.5264	0.4676	0.4858	0.4523	0.1446
node_14	0.2634	0.2582	0.1973	0.2725	0.2816	0.3574	0.3757	0.3275	0.2871	0.2978	0.2627	0.3217	0.3434	0.4525	0.3894	0.4182	0.3779	0.1207
**b — node_15**	0.5793	0.5323	0.3256	0.5657	0.4863	0.5854	0.5930	0.5018	0.5722	0.5692	0.5718	0.5753	0.5755	0.7862	0.6008	0.6577	0.5897	0.1941
node_16	0.2807	0.2833	0.2220	0.2809	0.2933	0.4812	0.4776	0.4101	0.3568	0.2896	0.2608	0.2785	0.4622	0.6450	0.4976	0.5442	0.4657	0.1589
**c1 — node_17**	0.1341	0.1357	0.1617	0.1330	0.1328	0.4145	0.4140	0.3400	0.1339	0.1342	0.1331	0.1337	0.3993	0.5377	0.4311	0.4552	0.3915	0.1333
**c2 — node_18**	0.1168	0.1176	0.1329	0.1178	0.1178	0.3221	0.3293	0.2634	0.1169	0.1165	0.1178	0.1171	0.3317	0.4766	0.3540	0.3776	0.3312	0.1165
node_19	0.0839	0.0856	0.0917	0.0835	0.0828	0.2023	0.2181	0.1683	0.0842	0.0840	0.0815	0.0854	0.2105	0.2866	0.2339	0.2407	0.2184	0.0748
node_20	0.0602	0.0619	0.0680	0.0603	0.0578	0.1447	0.1613	0.1283	0.0585	0.0610	0.0566	0.0617	0.1539	0.2046	0.1794	0.1821	0.1662	0.0573
(5) — node_21	0.5078	0.4662	0.2962	0.4714	0.4444	0.5268	0.5380	0.4640	0.5082	0.4755	0.4817	0.5036	0.5219	0.7188	0.5568	0.6011	0.5437	0.1781
(6) — node_22	0.4119	0.3958	0.2665	0.3968	0.3830	0.4557	0.4784	0.4138	0.4248	0.4086	0.4074	0.4364	0.4647	0.6291	0.5041	0.5351	0.4848	0.1594
node_23	0.3084	0.2961	0.2122	0.2894	0.2878	n.a.	0.3748	0.3221	0.3255	0.3091	0.3021	0.3068	0.3569	0.4673	0.3930	n.a.	n.a.	0.1291
node_24	0.3955	0.3696	0.2533	0.3685	0.3629	0.4312	0.4568	0.3939	0.4070	0.3890	0.3812	0.4071	0.4403	0.5940	0.4736	0.5084	n.a.	0.1519
node_25	0.3645	0.3425	0.2424	0.3425	0.3405	0.4121	0.4276	0.3712	0.3726	0.3512	0.3542	0.3764	0.4126	0.5552	0.4535	0.4890	0.4453	0.1434
(7) — node_26	0.3345	0.3151	0.2262	0.3163	0.3143	0.3848	0.3991	0.3448	0.3439	0.3231	0.3238	0.3499	0.3845	0.5057	0.4246	0.4574	0.4171	0.1335
node_27	0.2927	0.2788	0.2016	0.2801	0.2789	0.3402	0.3590	0.3093	0.3091	0.2860	0.2880	0.3118	0.3447	0.4517	0.3788	0.4073	0.3706	0.1196
node_28	0.2655	0.2542	0.1856	0.2536	0.2538	0.3131	0.3299	0.2846	0.2845	0.2601	0.2616	0.2820	0.3157	0.4145	0.3478	0.3755	0.3414	0.1099
node_29	0.2396	0.2304	0.1691	0.2284	0.2308	0.2843	0.3028	0.2643	0.2599	0.2344	0.2369	0.2569	0.2871	0.3754	0.3189	0.3444	0.3132	0.1008
node_30	0.2147	0.2061	0.1511	0.2048	0.2080	0.2571	0.2736	0.2408	0.2348	0.2075	0.2123	0.2300	0.2590	0.3379	0.2880	0.3125	0.2837	0.0912
Regression slope (*b*)	1	0.9794	0.6438	0.8491	0.9645	1.1162	1.0116	0.7353	1.0014	0.9832	0.9990	1.0131	1.0046	0.9349	0.8990	0.9035	0.8566	0.2723
r^2	1	0.9957	0.9683	0.9841	0.9911	0.9302	0.9500	0.9609	0.9959	0.9974	0.9940	0.9975	0.9555	0.8595	0.9628	0.9576	0.9649	0.9584

Prior for constraint: Uniform distribution (*uni*)

Node id	C7	C6 -a	C6 -b	C6 -c	C6 -d	C6 -e	C6 -f	C6 -g	C5 -bc	C4 -abc	C2 ab	C1 a	C1 b	C1 c
a	+		+	+	+	+	+	+	+		+	+		
b	+	+		+	+	+	+	+			+		+	
c1, c2	+	+	+		+	+	+	+						+
d	+	+	+	+		+	+	+	+	+				
e	+	+	+	+	+		+	+	+	+				
f	+	+	+	+	+	+		+	+	+				
g	+	+	+	+	+	+	+		+	+				
note:									out only	green only	red fossils			
**r — node_00**	2.8160	2.2045	2.6848	2.3742	2.4236	2.7204	2.5943	2.4866	2.6110	2.3009	2.3041	2.2675	2.0170	0.7693
node_01	1.0049	0.8440	0.9857	0.8492	0.8751	0.9451	0.9240	0.8880	0.9081	0.8333	0.8145	0.7881	0.6841	0.2675
**d — node_02**	0.8471	0.7067	0.8203	0.7205	0.7356	0.8004	0.7694	0.7458	0.7574	0.7029	0.6828	0.6634	0.5713	0.2248
**e — node_03**	0.6810	0.5755	0.6645	0.5868	0.6033	0.6431	0.6209	0.6054	0.6090	0.5670	0.5462	0.5337	0.4590	0.1805
**f — node_04**	0.5607	0.4889	0.5640	0.4876	0.5056	0.5343	0.5156	0.5071	0.5063	0.4682	0.4532	0.4456	0.3781	0.1483
**g — node_05**	0.2930	0.2374	0.2891	0.2524	0.2584	0.2680	0.2581	0.2470	0.2605	0.2377	0.2180	0.2291	0.1875	0.0753
**a — node_06**	1.9748	1.6030	1.9941	1.6390	1.7063	1.8507	1.7895	1.7235	1.8744	1.6150	1.5862	1.6138	1.4317	0.5470
node_07	1.5344	1.2186	1.5469	1.2170	1.2983	1.4680	1.4039	1.3942	1.4474	1.2223	1.2422	1.1544	1.1061	0.4204
node_08	1.3420	1.0758	1.3462	1.0631	1.1435	1.3067	1.2194	1.2293	1.2731	1.0800	1.0906	1.0055	0.9776	0.3677
node_09	1.8263	1.4897	1.8541	1.5153	1.5794	1.7015	1.6492	1.5931	1.7356	1.5072	1.4602	1.4957	1.3281	0.5081
(1) — node_10	1.6613	1.3790	1.6875	1.3681	1.4411	1.5306	1.5014	1.4469	1.5760	1.3758	1.3188	1.3629	1.2246	0.4647
(2) — node_11	1.3626	1.2061	1.3586	1.1366	1.1966	1.3179	1.2508	1.1596	1.2919	1.0847	1.1282	1.0957	0.9826	0.3664
(3) — node_12	1.1988	1.0496	1.2026	1.0021	1.0437	1.1610	1.0955	1.0217	1.1381	0.9564	0.9876	0.9547	0.8671	0.3256
(4) — node_13	0.7889	0.6586	0.7901	0.6698	0.6902	0.7599	0.7162	0.6982	0.7485	0.6351	0.6335	0.6453	0.5106	0.2142
node_14	0.6989	0.5770	0.6610	0.5722	0.6064	0.6378	0.6307	0.6061	0.6281	0.5560	0.5366	0.5434	0.4244	0.1791
**b — node_15**	1.0471	0.9226	1.0557	0.8864	0.9118	1.0158	0.9650	0.8996	0.9976	0.8365	0.8723	0.8353	0.7627	0.2845
node_16	0.8593	0.7489	0.8909	0.7385	0.7785	0.8766	0.8227	0.7479	0.8316	0.7224	0.7203	0.6925	0.6055	0.2292
**c1 — node_17**	0.7383	0.6537	0.7632	0.6444	0.6834	0.7407	0.6985	0.6322	0.7197	0.6049	0.5968	0.5723	0.5180	0.1915
**c2 — node_18**	0.6037	0.5208	0.6126	0.5037	0.5566	0.5925	0.5669	0.5111	0.5890	0.4875	0.4852	0.4637	0.4040	0.1613
node_19	0.3859	0.3208	0.4138	0.3332	0.3604	0.3916	0.3821	0.3224	0.3801	0.3096	0.3068	0.2975	0.2574	0.1054
node_20	0.2877	0.2340	0.3145	0.2545	0.2770	0.3027	0.2928	0.2367	0.2874	0.2291	0.2336	0.2306	0.1922	0.0787
(5) — node_21	0.9560	0.8433	0.9568	0.8166	0.8362	0.9221	0.8896	0.8218	0.9216	0.7653	n.a.	0.7645	n.a.	0.2601
(6) — node_22	0.8644	0.7413	0.8528	0.7200	0.7433	0.8444	0.7978	0.7636	0.8201	n.a.	n.a.	0.6706	n.a.	0.2316
node_23	0.6941	0.5784	0.6648	0.5711	n.a.	0.6701	0.6305	0.6072	0.6388	n.a.	n.a.	n.a.	n.a.	0.1808
node_24	0.8216	0.7054	0.8091	0.6822	n.a.	0.8043	0.7379	0.7275	0.7668	n.a.	0.6711	n.a.	n.a.	0.2198
node_25	0.7763	0.6625	0.7637	0.6416	0.6792	0.7565	0.7171	0.6884	0.7432	0.6228	n.a.	0.6029	n.a.	0.2068
(7) — node_26	0.7285	0.6171	0.7092	0.5954	0.6364	0.7103	0.6702	0.6449	0.6954	0.5842	0.6325	0.5613	n.a.	0.1928
node_27	0.6498	0.5505	0.6341	0.5317	0.5628	0.6334	0.6000	0.5767	0.6205	0.5200	0.5631	0.4952	0.5680	0.1715
node_28	0.5995	0.5055	0.5834	0.4907	0.5194	0.5809	0.5512	0.5325	0.5736	0.4791	0.5167	0.4544	0.5157	0.1586
node_29	0.5520	0.4618	0.5345	0.4516	0.4744	0.5329	0.5009	0.4866	0.5235	0.4425	0.4758	0.4148	0.4687	0.1452
node_30	0.4994	0.4183	0.4856	0.4085	0.4293	0.4830	0.4499	0.4376	0.4741	0.3999	0.4306	0.3734	0.4195	0.1305
Regression slope (*b*)	1.4936	1.1825	1.4632	1.2388	1.2695	1.4066	1.3544	1.3159	1.3978	1.2212	1.2054	1.2215	1.0799	0.4168
r^2	0.9581	0.9549	0.9505	0.9607	0.9546	0.9496	0.9531	0.9526	0.9527	0.9576	0.9575	0.9654	0.9568	0.9596

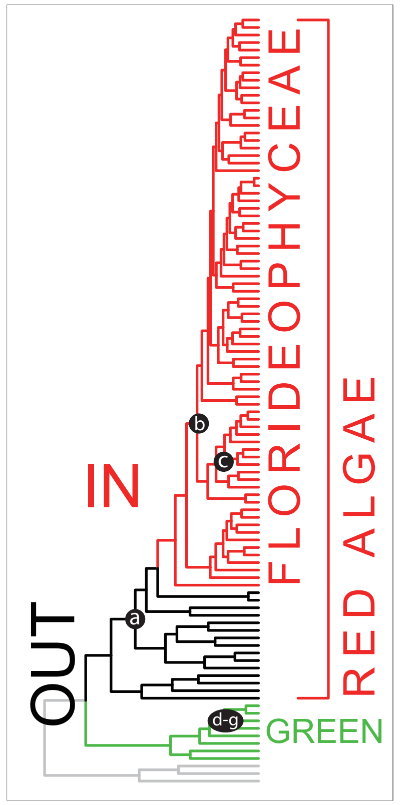

Regression function

Y = *a* + *b*X (Y, C7; X, CN)

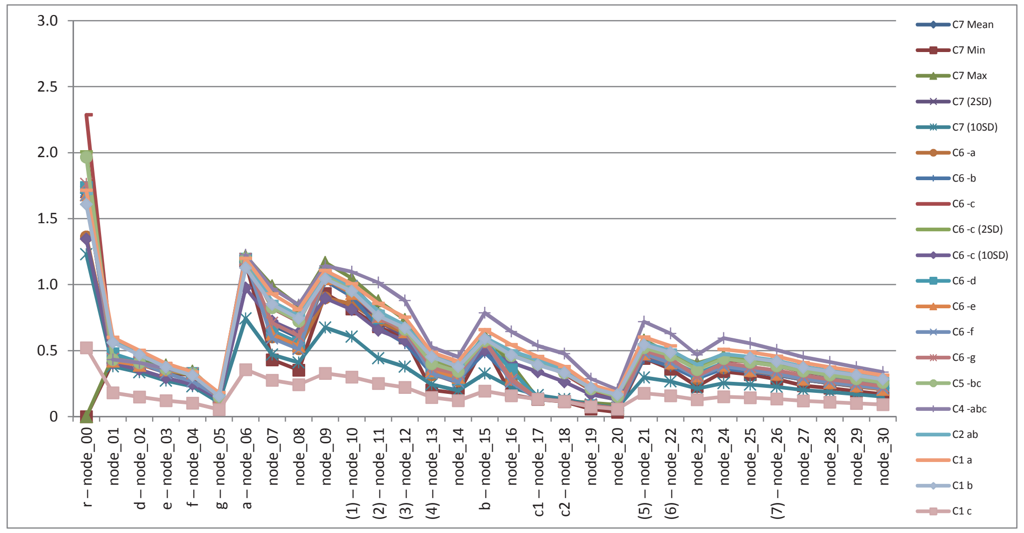

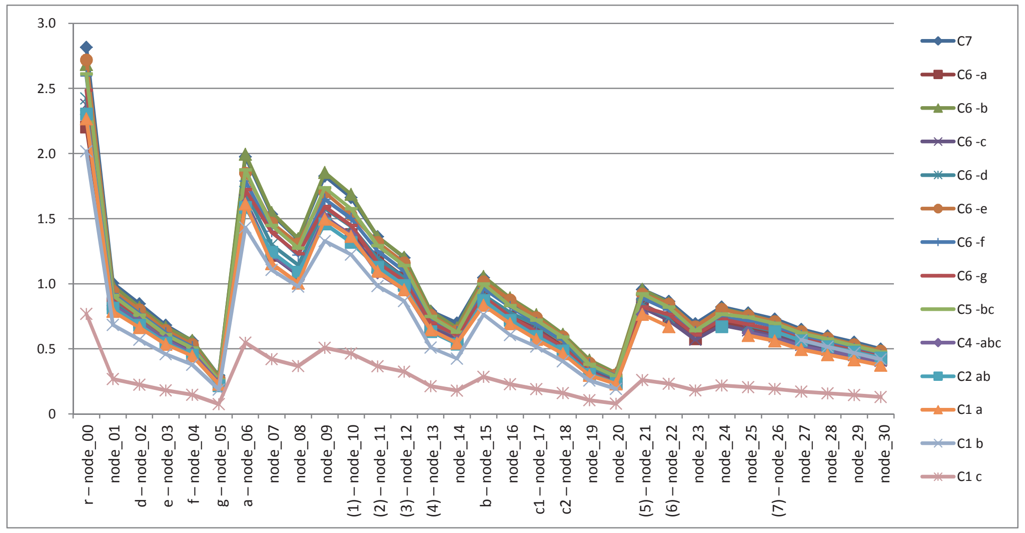

Supplementary Table S2. Time estimations comparison resulted in the BEAST analyses under different priors, i) Yule Process, ii) Birth-Death and iii) Birth-Death Incomplete process.

Prior for constraint:	Normal distribution (*nor*)		
	C7		
Node id	Yule	Birth-Death	Birth-Death Incomplete
**r — node_00**	1.6936	1.6715	1.6757
node_01	0.4341	0.4356	0.4365
**d — node_02**	0.4093	0.4097	0.4097
**e — node_03**	0.3611	0.3648	0.3633
**f — node_04**	0.3282	0.3271	0.3273
**g — node_05**	0.1503	0.1502	0.1504
**a — node_06**	1.1943	1.1942	1.1939
node_07	0.6938	0.6397	0.6315
node_08	0.5983	0.5529	0.5418
node_09	1.0596	1.0560	1.0673
(1) — node_10	0.9432	0.9172	0.9490
(2) — node_11	0.7805	0.7683	0.7605
(3) — node_12	0.6608	0.6510	0.6505
(4) — node_13	0.3312	0.3413	0.3545
node_14	0.2634	0.2773	0.2879
**b — node_15**	0.5793	0.5753	0.5751
node_16	0.2807	0.2671	0.2973
**c1 — node_17**	0.1341	0.1336	0.1334
**c2 — node_18**	0.1168	0.1172	0.1175
node_19	0.0839	0.0849	0.0849
node_20	0.0602	0.0611	0.0610
(5) — node_21	0.5078	0.4990	0.5048
(6) — node_22	0.4119	0.4159	0.4251
node_23	0.3084	0.3071	0.3179
node_24	0.3955	0.3869	0.3970
node_25	0.3645	0.3582	0.3692
(7) — node_26	0.3345	0.3315	0.3417
node_27	0.2927	0.2962	0.3035
node_28	0.2655	0.2697	0.2773
node_29	0.2396	0.2445	0.2518
node_30	0.2147	0.2196	0.2254
Regression slope (*b*)	1	1.0175	1.0163
r^2	1	0.9986	0.9976

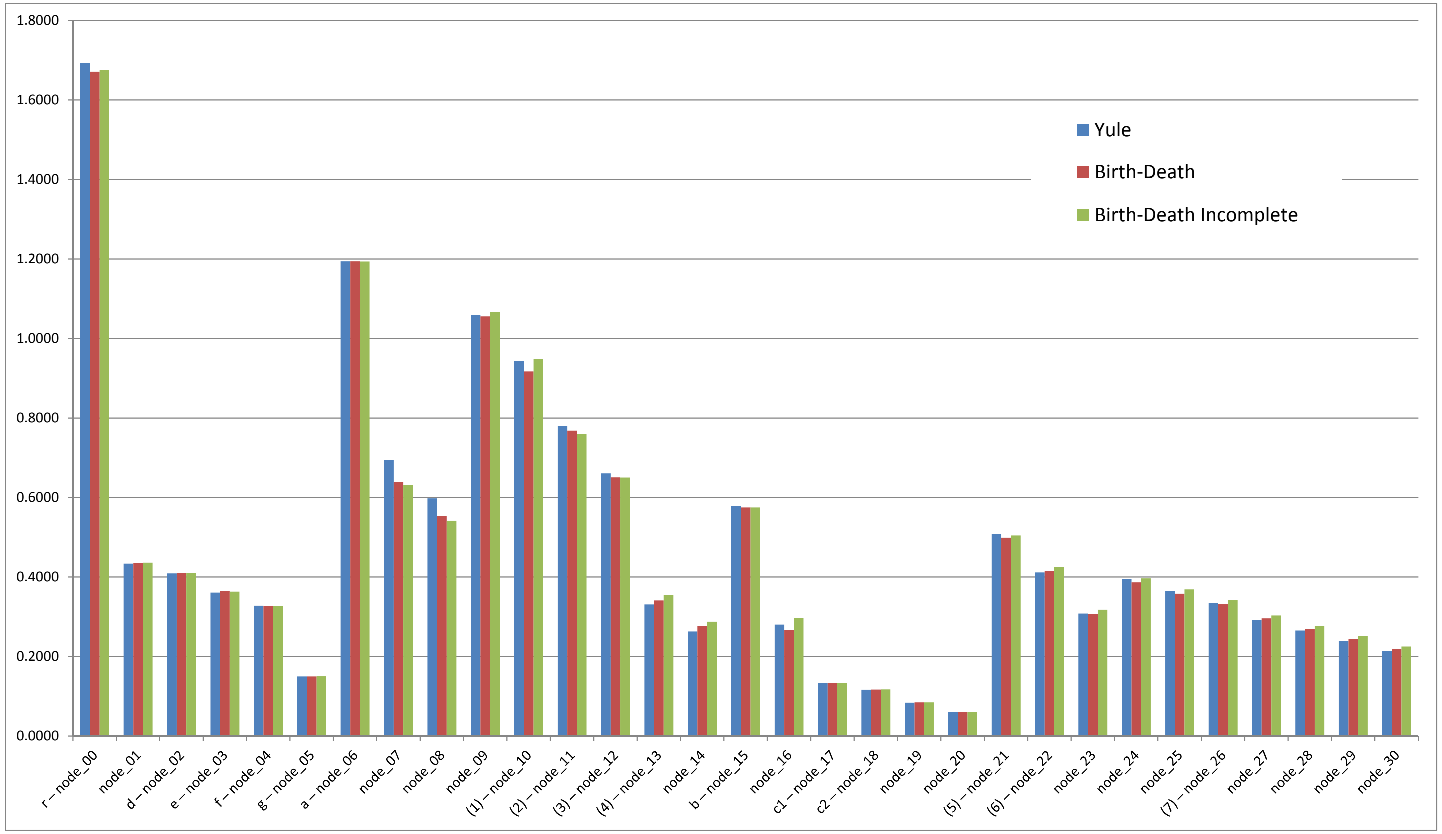

Table S3. Species examined in the present study. The sequences determined in this study are shown in bold text. Taxa name used in tree highlighted.

Taxa, collection details, and voucher number							
	Plastid			Nuclear			Mitochondria
	*psa*A	*psb*A	*rbc*L	EF-2	SSU	LSU	*cox*1
RHODOPHYTA							
BANGIOPHYCEAE							
Bangiales Nägeli							
Bangia atropurpurea (Roth) C. Agardh							
	AY119698	AY119734	AY119770	EF033517	D88387	AF419107	DQ442887
Porphyra purpurea (Roth) C. Agardh							
	NC_000925	NC_000925	NC_000925	EF033519	L26201	EF033596	NC_002007
COMPSOPOGONOPHYCEAE							
Compsopogonales Skuja							
Compsopogon caeruleus (Balbis ex C. Agardh) Montagne							
	AY119701	AY119737	AF087116	-	AF342748	-	-
Rhodochaetales Bessey							
Rhodochaete parvula Thuret							
	AY119707	AY119743	AY119777	-	AF139462	FJ973376	-
CYANIDIOPHYCEAE							
Cyanidiales T. Christensen							
Cyanidioschyzon merelae Luca, Taddei et Varano							
	AY119693	AY119693	AY119765	-	AB158485	AB158485	NC_000887
Cyanidium caldarium (Tilden) Geitler							
	NC_001840	NC_001840	NC_001840	-	AB090833	-	-
Cyanidium sp. Sybil Cave							
	AY391363	AY391366	AY391369	-	-	-	-
Galdieria sulphuraria (Galdieri) Merola							
	AY119695	AY119731	AY119767	-	AB091230	-	-
PORPHYRIDIOPHYCEAE							
Porphyridiales Kylin ex Skuja							
Flintiella sanguinaria Ott							
	AY119704	AY119740	AY119774	-	AY342749	-	-
Porphyridium aerugineum Geitler							
	AY119705	AY119741	AY119775	-	AF168623	-	-

Porphyridium cruentum (S.F.Gray) Nägeli						
EST_contig14145	EST_contig5624	EST_contig1070	EST_contig13758	-	-	EST_contig2709
RHODELLOPHYCEAE						
Rhodellales H.S. Yoon, K.M. Müller, R.G. sheath, F.D. Ott et D. Bhattacharya						
Dixoniella grisea (Geitler) Scott, Broadwater, Saunders, Thomas et Gabrielson						
AY119702	AY119738	AY119773	-	AB045581	-	-
Rhodella violacea (Kornmann) Wehrmeyer						
AY119706	AY119742	AY119776	-	EU861395	-	-
STYLONEMATOPHYCEAE						
Stylonematales Drew						
Rhodosorus marinus Geitler						
AY119708	AY119744	AY119778	-	AF342750	-	-
Stylonema alsidii (Zanardini) Drew						
AY119709	AY119745	AY119779	-	AF168633	-	-
FLORIDEOPHYCEAE						
Ahnfeltiophycidae						
Ahnfeltiales C.A. Maggs et C.M. Pueschel						
Ahnfeltia fastigiata (Linnaeus) J.V. Lamouroux; US: Alaska: Sitka: Kruzof Island (13 vii 2006); A313						
**FJ195596**	**FJ195601**	**FJ195605**	-	DQ343668	AF419104	GQ497301
Ahnfeltia plicata (Hudson) Fries						
-	-	-	EF033537	Z14139	AF419105	-
Corallinophycidae						
Corallinales P.C. Silva et H.W. Johansen (1986, p.250) emendavit W.A. Nelson, J.E. Sutherl., T.J. Farr et H.S. Yoon						
Amphiroa fragilissima (Linnaeus) J.V. Lamouroux						
-	-	U04039	EF033529	U60744	EF033599	-
Corallina spp. (*C. officinalis* + *C. pilulifera*)						
Corallina officinalis Linnaeus						
-	-	-	EF033530	L26184	AF419116	FM180081
Corallina pilulifera Postel et Ruprecht; Japan: Chiba: Choshi (01 viii 2004); C1003						
**DQ787594**	**DQ787634**	**DQ787558**	-	-	-	-
Mastophora rosea (C. Agardh) Setchell; USA: Guam: Pago Bay; PAGO0003B; RedToL #538						
**KP224281**	**KP224287**	**KP224278**	**KP224279**	**KP224295**	**KP224296**	**KP224298**
Jania sagittata (Lamouroux) Blainville; Australia: Tasmania: Stanley Breakwater; GWS016470; RedToL #536						
**KP224282**	**KP224288**	KC134331	KC130175	KC157580	KC157591	**KP224299**
Pseudolithophyllum muricatum (Foslie) Steneck et R.T. Paine						
-	-	AY294373	-	-	-	
Hapalidiales W.A. Nelson, J.E. Sutherl., T.J. Farr et H.S. Yoon						

Mesophyllum vancouveriense (Foslie) Steneck et R.T. Paine; Canada: British Columbia: Tahsis; GWS010090; RedToL #542						
-	**KP224289**	KC134326	KC130171	KC157577	KC157589	**KP224300**
Lithothamnion glaciale Kjellman; Canada: Newfoundland and Labrador: Maerl bed; GWS007312; RedToL #545						
-	**KP224290**	KC134336	KC130177	-	KC157593	HM918805
Synarthrophyton patena (J.D. Hooker et Harvey) R.A. Townsend; Australia: Victoria: South of Queenscliff Pier; KRD833; RedToL #547						
**KP224283**	**KP224291**	KC134328	EF033531	KC157578	EF033600	**KP224302**
Rhodogorgonales S. Fredericq, J.N. Norris et C. Pueschel						
Renouxia antillana Fredericq et J.N. Norris						
-	-	U04181	-	-	-	
-	-	-	EF033534	EF033584	EF033601	GQ497316
Rhodogorgon ramosissima J.N. Norris et Bucher						
-	-	U04183	EF033535	AF006089	EF033602	-
Sporolithales L. Le Gall et G.W. Saunders						
Heydrichia homalopasta R.A. Townsend et Borowitzka						
-	DQ167931	-	-	AF411629	-	-
Sporolithon durum (Foslie) R.A. Townsend et Woelkerling						
-	KC963421	-	-	AF411626	-	NC_023454
Sporolithon ptychoides Heydrich						
-	KC870926	-	GQ149067	GQ149066	GQ149068	HQ422711
Hildenbrandiophycidae						
Hildenbrandiales Pueschel et Cole						
Hildenbrandia rubra (Sommerfelt) Meneghini; CCAP 1368/2						
**DQ787593**	**DQ787633**	**DQ787557**/AF208801	EF033522	AF076995	AF419136	GQ497309
Nemaliophycidae						
Acrochaetiales Feldmann						
Acrochaetium spp. (*A. savianum* + *A. secundatum* + *Acrochaetium* sp.)						
Acrochaetium savianum (Meneghini) Nägeli; CCAP 1350/2						
**DQ787597**	**DQ787637**	**DQ787561**	-	-	-	-
Acrochaetium secundatum (Lyngbye) Nägeli						
-	-	-	EF033523	AF079784	AF528044	-
Acrochaetium sp.						
-	-	-	-	-	-	GQ497300
Balbianiales R.G. Sheath et K.M. Müller						
Balbiania investiens (Lenormand ex Kützing)						
-	-	AF132293	EF033524	AF132294	AF421124	-
Balliales H.-G. Choi, Kraft et G.W. Saunders						
Ballia callitricha (C. Agardh) Kützing; New Zealand: Marlborough: Kaikoura (20 viii 2002); B10						
**DQ787595**	**DQ787635**	**DQ787559**	EF033525	AF236790	AF419106	-

Batrachospermales Pueschel et K.M. Cole						
Batrachospermaceae spp. (*B. gelatinosum* + *P. bernabei*)						
Batrachospermum gelatinosum (Linnaeus) De Candolle; UTEX LB1493						
**DQ787596**	**DQ787636**	**DQ787560**	-	AF026045	DQ903115	EU636744
Petrohua bernabei G.W. Saunders						
-	-	-	EF033526	-	-	-
Colaconematales J.T. Harper et G.W. Saunders						
Colaconema caespitosum (J. Agardh) Jackelman, Stegenga et J.J. Bolton; Australia: Victoria: Point Lonsdale Lighthouse Reef; GWSC3582; RedToL #557						
**KP224284**	**KP224292**	KC134354	EF033528	AF079787	AF528046	**KP224303**
Colaconema proskaueri (West) P.W. Gabrielson; Canada: British Columbia: Banfield Bradys Beach; GWSC008; RedToL #559						
**KP224285**	**KP224293**	KC134327	KC130172	AF079791	AF528049	KF364496
Nemaliales Schmitz						
Nemalion spp. (*N. helminthoides* + *Nemalion* sp. N10 + *Nemalion* sp.)						
Nemalion helminthoides (Velley in Withering) Batters						
-	-	-	EF033532	L26196	AY570376	-
Nemalion sp.; England: Devon: Wembury Beach (28 vii 2003); N10						
**DQ787598**	**DQ787638**	**DQ787562**	-	-	-	-
Nemalion sp.						
-	-	-	-	-	-	GQ497310
Palmariales Guiry et D. Irvine						
Palmaria palmata (Linnaeus) Kuntze; Northern Ireland: Noyle: Garron (24 vii 2003); P244						
**DQ787599**	**DQ787639**	**DQ787563**	EF033533	Z14142	Y11506	GQ497313
Rhodachlyales G.W. Saunders, S.L.Clayden, J.L. Scott, K.A. West, U. Karsten et J.A. West						
Rhodachlya madagascarensis J.A. West, J.L. Scott, K.A. West, U. Karsten, S.L. Clayden & G.W. Saunders; Madagascar: Ifaty; JAW4326; RedToL # 588						
**KP224286**	**KP224294**	KC134337	EU262262	EU262260	EU262261	**KP233841**
Thoreales Müller, Sheath, Sherwodd et Pueschel						
Thorea spp. (*T. violacea* + Thoreaceae sp.)						
Thorea violacea Bory de Saint-Vincent						
AY119712	AY119747	AF029160	-	-	-	-
Thoreaceae sp.						
-	-	-	EF033536	AF420253	AF419145	-
Rhodymeniophycidae						
Acrosymphytales R.D. Withall et G.W. Saunders						
Acrosymphyton caribaeum (J. Agardh) G. Sjostedt						
**KM359975**	**KM359999**	**KM360022**	EF033539	DQ343661	DQ343684	-
Schimmelmannia schousboei (J. Agardh) J. Agardh						
**KM359976**	**KM360000**	**KM360023**	EF033540	AY437681	AF419130	-
Bonnemaisoniales Feldmann et Feldmann						

Bonnemaisonia hamifera hariot; Japan: Hokkaido: Muroran (25 iv 2002); B8							
	**FJ195594**	**FJ195600**	**FJ195604**	AY010232	L26182	AF419112	-
Delisea hypneoides Harvey							
	**KM359977**	-	**KM360024**	EF033541	EF033585	EF033603	-
Naccariaceae spp. (*Naccaria wiggii* + *Reticulocaulis mucosissimus*)							
Naccaria wiggii (Turner) Endlicher							
	-	-	-	-	-	-	GQ497312
Reticulocaulis mucosissimus Abbott							
	**KM359978**	**KM360001**	**KM360025**	EF033542	DQ343656	DQ343680	-
Ceramiales Oltmanns							
Callithamnion spp. (*C. collabens* + *C. onsanguineum*)							
Callithamnion collabens (Rudolphi) McIvor et Maggs							
	-	-	-	-	DQ022769	DQ022974	-
Callithamnioin consanguineum J.D. Hooker et Harvey; New Zealand: Wellington: Breaker Bay (11 ix 2002); C309							
	**DQ787602**	**DQ787642**	**DQ787565**	-	-	-	EU194962
Seirospora interrupta (Smith) F. Schmitz							
	**DQ787615**	**DQ787649**	DQ110903	-	DQ022774	DQ022799	EU194970
Centroceras clavulatum (C. Agardh) Montagne							
	AY295137	AY178488	AY295175	EF033543	DQ343657	AF419113	EU194971
Ceramium spp. (*C. secundatum* + *C. virgatum*)							
Ceramium secundatum Lyngbye; France: Roscoff (05 iv 2000); C197							
	**DQ787605**	**DQ787644**	DQ110904	-	-	-	EU194972
Ceramium virgatum Roth							
	-	-	-	EF033544	AF236793	EF033604	-
Griffithsia spp. (*G. corallinoides* + *G. okiensis*)							
Griffithsia corallinoides (Linnaeus) Trevisan							
	AY295126	AY295146	AY295164	-	EU718690	-	-
Griffithsia okiensis Kajimura							
	-	-	-	-	-	-	EU194973
Ptilota spp. (*P. gunneri* + *P. serrata*)							
Ptilota gunneri P.C. Silva, Maggs et L.M. Irvine; Northern Ireland: Moyle: Cushendun (24 iiv 2003); P386							
	**DQ787613**	AY865154	**DQ787575**	-	EU718700	-	EU194975
Ptilota serrata Kützing							
	-	-	-	EF033545	-	EF033605	-
Spyridia filamentosa (Wulfen) Harvey; Korea: Pohang: Wolpo (16 xi 2002); S16							
	**DQ787618**	**DQ787652**	**DQ787579**	-	EU718707	AF458717	AF458719
Dasyaceae spp. (*D. collabens* + *H. plumosa*)							
Dasya collabens J.D. Hooker et Harbey; Korea: Gangreung: Anin (30 I 2002); D4							

**DQ787619**	**DQ787653**	**DQ787580**	-	AF488384	-	-
Heterosiphonia plumosa (Ellis) Batters						
-	-	-	EF033546	-	EF033606	
Dasysiphonia spp. (*D. chejuensis* + *D. okiensis*)						
Dasysiphonia okiensis Kajimura; Japan: Oki Island: Kamo Bay (06 v 2003); D56						
**DQ787620**	**DQ787654**	**DQ787581**	-	-	-	GQ497304
Dasysiphonia chejuensis I.K. Lee et W.A. West						
-	-	-	-	AF488388	-	-
Delesseriaceae spp. 1 (*Delesseria serrulata* + *Grinnellia americana*)						
Delesseria serrulata Harvey; Korea: Gangreung: Anin (30 i 2002); D19						
**DQ787621**	**DQ787655**	**DQ787582**	-	AF488403	-	-
Grinnellia americana (C. Agardh) Harvey						
-	-	-	EF033547	-	EF033607	-
Delesseriaceae spp. 2 (*Phycodrys rubens* + *Sorella repens* + *Hymenena* sp.)						
Phycodrys rubens (Linnaeus) Batters; Norway: Møre og Romsdal: Ona Island (28 vii 2004); P422						
**DQ787622**	**DQ787656**	**DQ787583**	-	-	-	-
Sorella repens (Okamura) Hollenberg						
-	-	-	EF033548	AF488406	EF033608	-
Hymenena sp.						
-	-	-	-	-	-	GQ497305
Brongniartella byssoides (Goodenough et Woodward) F. Schmitz: Norway: Møre og Romsdal: Pinnøya (29 vii 2004); B19						
**DQ787623**	**DQ787657**	**DQ787584**	-	-	-	-
Chondrophycus intermedius (Yamada) Garbary et Harper; Korea: Gangwon: Goseong: Ayajin (30 vii 2002); L2						
**DQ787624**	**DQ787658**	**DQ787585**	-	-	-	-
Polysihponia spp. (*P. stricta* + *Polysiphonia* sp.)						
Polysiphonia stricta (Dillwyn) Greville; Norway; Møre og Romsdal: Pinnøya (29 vii 2004); P421						
**DQ787625**	**DQ787659**	AY958166	-	AF427535	-	-
Polysiphonia sp.						
-	-	-	-	-	-	GU385828
Gelidiales Kylin						
Gelidium spp. (*G. elegans* + *G. australe* + *G. purpurascens*)						
Gelidium elegans (J.V. Lamouroux) J.V. Lamouroux; Korea: Jejudo: Seongsan (23 vii 2002); G2						
**DQ787626**	**DQ787660**	**DQ787586**	-	-	-	-
Gelidium australe J. Agardh						
-	-	-	EF033549	DQ343660	DQ343682	-
Gelidium purpurascens N.L. Gardner						
-	-	-	-	-	-	GQ497307
Gigartinales Schmitz						

Ahnfeltiopsis spp. (*A. paradoxa* + *A. linearis*)						
Ahnfeltiopsis paradoxa (Suringar) Masuda; Japan: Chiba: choshi (27 vii 2002); A12						
**DQ787627**	**DQ787661**	**DQ787587**	-	-	-	-
Ahnfeltiopsis linearis (C. Agardh) P.C. Silva et DeCew (GWS002887)						
-	-	-	-	-	GQ338104	GQ380028
Chondrus crispus Stackhouse; GWS009227						
**KM359979**	**KM360002**	**KM360026**	EF033554	Z14140	AF419120	NC_001677
Euthora cristata (C. Agardh) J. Agardh; GWS000026 (G0433)						
**KM359980**	**KM360003**	**KM360027**	EF033555	AY437684	AF419124	GU140142
Gloiopeltis furcata (Postels et Ruprecht) J.G. Agardh; GWS002264						
**KM359981**	**KM360004**	**KM360028**	EF033553	U33130	EF033612	-
Hypnea spp. (*H. japonica* + *H. charoides*)						
Hypnea japonica Tanaka; Korea: Ulreungdo: Tonggumi (27 viii 2003); H41						
**FJ195597**	DQ095851	DA095829	-	-	-	EU345986
Hypnea charoides J.V. Lamouroux						
-	-	-	-	AY437682	-	-
Mychodea acanthymenia Kraft; GWS000960						
**KM359982**	**KM360005**	**KM360029**	EF033557	EF033589	EF033614	-
Neodilsea borealis (Abbott) Lindstrom; G0223						
**KM359983**	**KM360006**	**KM360030**	EF033550	AF317112	EF033610	EU189312
Weeksia coccinea (Harvey) Lindstrom; GWS001705						
**KM359984**	**KM360007**	**KM360031**	EF033552	AF317120	EF033611	EU189325
Gracilariales S. Fredericq et M.H. Hommersand						
Gracilaria tenuistipitata var. liui Zhang et Xia						
AY673996	AY673996	AY673996	-	DQ316995	-	EF434924
Gracilaria spp. (*G. textorii* + *G. salicornia*)						
Gracilaria textorii De Toni; Korea: Chungnam: Daecheon (17 i 2000); G45						
**FJ195595**	DQ095832	DQ095793	-	-	-	EF434925
Gracilaria salicornia (C. Agardh) E.Y. Dawson						
-	-	-	EF033559	AY204142	EF033615	-
Gracilariopsis spp. (*G. chorda* + *G. andersonii* + *G. lemaneiformis*)						
Gracilariopsis chorda (Holmes) Ohmi; Korea: Jindo: Hoidong (09 iii 2001); G39						
-	DQ095825	DQ095785	-	-	-	EU567358
Gracilariopsis andersonii (Grunow) Dawson; GWS002277						
-	-	-	EF033560	-	-	-
Gracilariopsis lemaneiformis (Bory de Saint-Vincent) E.Y. Dawson, Acleto et Foldvik						
-	-	-	-	L26214	AF419132	-
Halymeniales G.W. Saunders et G.T. Kraft						

Grateloupia spp. (*G. divaricata* + *G. subpectinata* + *Grateloupia* sp.)						
Grateloupia divaricata Okamura; Korea: Taean: Hakampo (11 xi 2001); G50						
**DQ787630**	**DQ787664**	**DQ787590**	-	-	-	-
Grateloupia subpectinata Holmes						
-	-	-	-	U33132	-	-
Grateloupia sp. GWS003281						
-	-	-	-	-	-	GQ497308
Epiphloea bullosa (Harvey) De Toni; GWS001054						
**KM359985**	**KM360008**	**KM360032**	EF033562	AY437701	DQ343693	-
Isabbottia ovaliforlia (Kylin) Balakrishnan; GWS001334						
**KM359986**	**KM360009**	**KM360033**	EF033563	EF033590	EF033616	-
Halymeniaceae spp. (*P. carnosa* + *H. pseudofloresii*)						
Pachymenia carnosa (J. Agardh) J. Agardh; GWS000347						
**KM359987**	**KM360010**	**KM360034**	EF033564	AF515289	DQ343695	-
Halymenia pseudofloresii F.S. Collins et M.A. Howe; CL033701						
-	-	-	-	-	-	GQ862076
Zymurgia chondriopsidea (J. Agardh) Lewis et Kraft; GWS000966						
**KM359988**	**KM360011**	**KM360035**	EF033566	AF515304	DQ343698	-
Nemastomatales Kylin						
Predaea kraftiana Millar et Guiry; GWS000132						
**KM359989**	**KM360012**	-	EF033567	AF515297	EF033618	-
Platoma cyclocolpum (Montagne) Schmitz; GWS011425						
**KM359990**	**KM360013**	**KM360036**	EF033568	AF515292	DQ343706	-
Schizymenia pacifica (Kylin) Kylin; GWS001682						
**KM359991**	**KM360014**	**KM360037**	EF033569	EF033592	AF419129	GQ497319
Peyssonneliales D.M. Krayesky, Fredericq et J.N. Norris						
Ramicrusta textilis C.M. Pueschel et G.W. Saunders; GWS001755						
**KM359992**	**KM360015**	**KM360038**	-	FJ848977	FJ848970	-
Sonderopelta coriacea Womersley et Sinkora; GWS001476						
**KM359993**	**KM360016**	**KM360039**	EF033558	AY437689	DQ343691	-
Plocamiales G.W. Saunders et G.T. Kraft						
Plocamium spp. (*P. cartilagineum* + *P. maggsiae* + *Plocamium* sp.)						
Plocamium cartilagineum (Linnaeus) P.S. Dixon; Korea: Pohang: Guryeongpo (31 i 2002); P13						
**DQ787629**	**DQ787663**	**DQ787589**	-	-	-	
Plocamium maggsiae G.W. Saunders et Lehmkuhl						
-	-	-	EF033570	AY437708	AF419141	
Plocamium sp. LLG0171						
-	-	-	-	-	-	GQ497314

Sarcodia spp. (*S. ciliata* + *Sarcodia* sp. GWS002597)						
Sarcodia ciliata Zanardini; GWS001027						
**KM359994**	**KM360017**	**KM360040**	EF033572	DQ343666	DQ343708	
Sarcodia sp. GWS002597						
-	-	-	-	-	-	FJ499623
Rhodymeniales Schmitz						
Champia spp. (*C. chathamensis* + *C. affinis* + *C. gigantea*)						
Champia chathamensis V.J. Chapman et Dromgoole; New Zealand: Wellington: Lyall Bay (03 viii 2001); C255						
**FJ195598**	**FJ195602**	**FJ195606**	-	-	-	-
Champia affinis (j.d. Hooker et Harvey) Harvey						
-	-	-	-	U23951	-	-
Champia gigantea M.J. Wynne						
-	-	-	EU624164	-	EU624159	-
Fryeella gardneri (Setchell) Kylin; GWS001131						
**KM359996**	**KM360019**	**KM360042**	EF033578	AF085273	EF033622	GQ497306
Gloioderma halymenioides (Harvey) J. Agardh; GWS000469						
**KM359995**	**KM360018**	**KM360041**	EF033574	DQ873283	DQ873283	-
Lomentaria spp. (*L. catenata* + *L. australis* + *Lomentaria* sp. GWS001885)						
Lomentaria catenata Harvey; Korea: Gyeongnam: Sacheon (18 vii 2001); L76						
**FJ195599**	**FJ105603**	**FJ195607**	EU624178	-	EU624155	-
Lomentaria australis (Kützing) Levring						
-	-	-	-	U33134	-	-
Lomentaria sp. GWS001885						
-	-	-	-	-	-	GQ497311
Rhodymenia intricata (Okamura) Okamura; Korea: Ulreungdo: Dodong (26 viii 2003); R13						
**DQ787631**	**DQ787665**	**DQ787591**	EU624196	AB381929	EU624150	-
Sebdeniales R.D. Withall et G.W. Saunders						
Lesleigha sp. 1LH; GWS002089						
**KM359997**	**KM360020**	**KM360043**	EF033581	AY437707	DQ343700	-
Sebdenia spp. (*S. flabellata* + GWS002074)						
Sebdenia flabellata (J. Agardh) P.G. Parkinson						
-	-	-	EF033579	U33138	AF419134	-
Sebdeniaceae sp. Unknown; GWS002074						
**KM359998**	**KM360021**	**KM360044**	-	-	-	-
CHAROPHYTA						
MESOSTIGMATOPHYCEAE						
Chaetosphaeridium globosum (Nordstedt) Klebahn						

NC_004115	NC_004115	-	-	AJ250110	-	NP_689386
Mesostigma viride Lauterborn						
NC_002186	NC_002186	-	EU812184/ EU812185	AJ250109	AY591912	NC_008240
CHLOROPHYTA						
TREBOUXIOPHYCEAE						
Chlorella vulgaris Beijerinck						
NC_001865	NC_001865	-	-	GQ122334	AB237642	AB011523
STREPTOPHYTA						
Arabidopsis thaliana (L.) Heynhold						
NC_000932	NC_000932	-	AC009894	NR_022795	AC006837	NC_001284
Marchantia polymorpha L.						
NC_001319	NC_001319	-	-	AY342318	AY342318	NC_001660
Pinus spp. (*P. thunbergii* + *P. wallichiana* + *P. kwangtungensis*)						
Pinus thunbergii Parl.						
NC_001631	NC_001631	-	-	-	-	-
Pinus wallichiana A.B. Jacks						
-	-	-	-	X75080	AJ271114	-
Pinus kwangtungensis = *Pinus fenzeliana* Hand.-Mazz.						
-	-	-	-	-	-	EF114116
Psilotum nudum (L.) Beauvois						
NC_003386	NC_003386	-	-	X81963	EU161326	EU161444
Zea mays L.						
NC_001666	NC_001666	-	EU970836	AF168884	AJ309824	AY506529
Outgroup						
CYANOBACTERIA						
CYANOPHYCEAE						
Nostoc sp. PPC 7120						
NC_003272	NC_003272	-	-	-	-	NC_003272
Synechocystis sp. PCC 6803						
NC_000911	NC_000911	-	-	-	-	NC_000911
Thermosynechococcus elongatus BP-1						
NC_004113	NC_004113	-	-	-	-	NC_004113
